
JOURNAL INFORMATION
==============================
NLM Title Abbreviation: J Orthop Traumatol
Journal ID: J Orthop Traumatol
NLM Journal ID: 101090931

Journal of Orthopaedics and Traumatology : Official Journal of the Italian Society of Orthopaedics and Traumatology

ISSN: 1590-9921
EISSN: 1590-9999
Publisher: Springer

ARTICLE INFORMATION
==============================
PMCID: PMC4417746
DOI: 10.1007/s10195-011-0148-9
Article ID: 148
Article version: 1
Subjects: Events

Oral Communications

Electronic publication date: 2011 Sep 2
Print publication date: 2011 Oct
Volume: 12
Issue: Suppl 1
First page: 1
Last page: 22
Copyright: © The Author(s) 2011
License: Open AccessThis article is distributed under the terms of the Creative Commons Attribution 2.0 International License (https://creativecommons.org/licenses/by/2.0), which permits unrestricted use, distribution, and reproduction in any medium, provided the original work is properly cited.
License URL: https://creativecommons.org/licenses/by/2.0/

Keywords: Rotator Cuff, Humeral Head, Radial Head, Intramedullary Nail, Suture Anchor
issue-copyright-statement: © The Author(s) 2011

==============================
J Orthopaed Traumatol (2011) 12 (Suppl 1):S1–S22
DOI 10.1007/s10195-011-0148-9

1 OCTOBER 2011

### Oral Communications

### ORAL COMMUNICATIONS

### C01—MUSCULOSKELETAL TUMORS AND METABOLIC DISEASES 1

#### Vascularized fibular autografts in upper limb oncological reconstructions

D.A. Campanacci*[1], D. Matera[1], F. Frenos[1], G. Caff[1], G. Beltrami[1], G. Scoccianti[1], L. Del Croix[2], M. Innocenti[2], R. Capanna[1]

[1]S.O.D. di Ortopedia Oncologica e Ricostruttiva, Azienda Ospedaliera Universitaria Careggi (Florence, IT);
[2]S.O.D. di Microchirurgia, Azienda Ospedaliera Universitaria Careggi (Florence, IT)

**Introduction** The objective of the present study was to review our series of vascularized fibular graft (VFG) reconstructions in intercalary and articular bone defects of the upper limb after oncological resection.
**Materials and methods** The total number of patients was divided in 4 groups concerning reconstruction type: 13 intercalary (11 VFG, 2 VFG + allograft); 12 growth plate transplantation (8 proximal humerus, 4 distal radius); 6 wrist arthrodesis; 2 proximal humerus reconstruction with allograft + VFG. We observed 15 complications at recipient site requiring surgical revision in 8 cases with implant removal in 3 cases. A fibular fracture occurred in 11 cases, healed after conservative treatment in 7 cases and after new osteosynthesis in 2, while in 2 cases the graft was removed and replaced with a prosthetic implant. Deep infection occurred in 1 case requiring graft removal. At donor site, in growth plate transplantation 60% of patients presented a peroneal nerve deficit, spontaneously resolved in all but 1 case. One patient developed a valgus ankle deformity.
**Results** At a mean follow-up of 73.5 months (1–165), 29 patients were continuously disease free, 3 patients were alive after metastasectomy and 2 patients were died of the disease. Functional results according with MSTS on 28 evaluable patients were excellent in 20 cases, good in 6 and fair in 2 cases.
**Conclusions** VFG showed to be a valid reconstructive option after bone tumor resections of the upper limb. In intercalary resections, a stable and long lasting reconstruction was achieved with only one failure due to deep infection. Wrist arthrodesis showed 100% success rate at long term with satisfactory functional results. Growth plate transplantation in children allowed excellent function but was technically demanding, presenting the risk of donor site complications. The association of proximal humerus osteoarticular allograft and vascularized fibula was unsuccessful resulting in a failure for fracture in both patients.

### C02—MUSCULOSKELETAL TUMORS AND METABOLIC DISEASES 2

#### Spine osteoblastoma: staging, treatment and outcome

R. Ghermandi*[1], S. Bandiera[1], A. Gasbarrini[1], G. Barbanti Bròdano[1], S. Terzi[1], L. Boriani[1], A. Corghi[1], S. Colangeli[1], C. Simoes[1], L. Babbi[1], F. Santoro[1], S. Boriani[1]

[1]Istituto Ortopedico Rizzoli (Bologna, IT)

**Introduction** Osteoblastoma (OBL) is a rare and benign osteoid producing primary bone tumor. Spine location is observed in 36% of these tumors, and the vast majority arises from the posterior elements. The issues of distinguishing from Osteoid osteoma are the size (>2 cm), spectrum of clinical behavior, local aggressiveness and its capacity of malignant transformation. The treatment of this tumor is a challenge and different options are described in literature. The aim of this paper is to evaluate retrospectively the results obtained with the surgical treatment planned following the Enneking Staging System.
**Materials and methods** From 1984 to 2010, 51 patients (34 male, 17 female) affected by spine OBL, average age 24.1 (range 7–60 years), average follow up 90 months (range 25–229 months). Following the Enneking Staging System, 10 cases (20%) were classified as Stage 2, 41 cases (80%) as Stage 3. The tumor extension was evaluated following the WWB system. The neurological status was evaluated following the Frankel classification (48 E, 2 D3, 1 D2). After 1998 in case of a planned intralesional excision a preoperative selective arterial embolisation was performed. All the patients underwent a surgical procedure planned following the Enneking Staging System (38 intralesional excision, 13 en bloc resection). In 5 cases (Stage 3 OBL) the intralesional excision was followed by Radiation Therapy in order to complete the treatment.
**Results** 7 local recurrence (LR), St. 3 OBL in all cases: 2 after en bloc resection, 5 after intralesional excision. Five cases out of 7 were non intact cases (patient already treated in other hospital).
**Discussion** A review of the literature shows that in case of incomplete resection, the local recurrence is more than 50% in case of St. 3 OBL and about 10%–15% in case of St. 2 OBL. That means that the surgical procedure must be planned after an accurate evaluation of the tumor stage. The role of radiation therapy in recurrent lesions or lesions that are incompletely resected is controversial, with the majority of cases illustrating no advantage.
**Conclusions** The first treatment significantly affects the prognosis. Following the Enneking Stagin System intralesional excision proved to be effective in stage 2 lesions and en bloc resection in stage 3 lesions. Radiation therapy represents a treatment option in case of huge tumors located in difficult surgical areas and in recurrent or inappropriately removed tumors.

### The role of the selective arterial embolization for the treatment of aneurismal bone cyst of the spine: a prospective study

R. Ghermandi*[1], S. Bandiera[1], A. Gasbarrini[1], G. Barbanti Bròdano[1], S. Terzi[1], L. Boriani[1], A. Corghi[1], S. Colangeli[1], L. Babbi[1], F. Santoro[1], S. Boriani[1]

[1]Istituto Ortopedico Rizzoli (Bologna, IT)

**Introduction** Aneurismal Bone Cyst (ABC) is a benign cystic lesion of the bone made of blood containing gaps separated by connective tissue. In 10–30% of cases ABC is localized at spine level. To this day different treatment options exist: (1) curettage and bone grafting; (2) en bloc excision; (3) selective arterial embolization (SAE); (4) radiotherapy; (5) combination of these procedures. The aim of this paper is to evaluate in a prospective manner the results of ABC treatment by SAE alone.

**Materials and methods** Seven patients (4 female, 3 male) with primary Aneurismal Bone Cyst of the spine in the absence of instability were analyzed. Average age was 19.6 years (range: 12–42 years) and average follow up period was 28.7 months (range: 9.4–49.4 months). Patients underwent the same diagnostic protocol (X-ray, CT scan, MRI) and the same treatment (selective arterial embolization) for different times depending on the cases. The same evaluation protocol was used for all patients (X-ray and CT scan at 8 weeks following embolization).

**Results** Patients performed from one to seven embolization treatments. No local recurrence or disease progression was recorded. No surgical treatment was required. For one patient, having a lesion at C7 level, an Halo Vest was necessary for 30 days, being substituted by a Minerva plaster for 3 months. All the patients were with no evidence of disease (NED) at follow up visits. The mean length of hospital stay was 2.2 days for each embolization treatment (range 2–5 days).

**Discussion** Even if it is a benign tumor, ABC can be locally aggressive and cause pain, functional impairment and pathological fractures. At spine level this lesion can involve mechanical compression of nerve roots or spinal cord with consequent generation of sensory-motor deficits. Initially, embolization was used as adjuvant procedure to reduce bleeding during surgery. First, De Rosa in 1990 treated ABC with embolization alone. Results from the literature demonstrate that the SAE efficacy is comparable only to that of surgery.

**Conclusions** SAE represents an effective, safe and minimally invasive approach for the treatment of ABC if it is performed by an interventional radiologist having specific preparation and experience. It should be the elective treatment for ABC in the absence of instability or serious neurological complications. In the case of failure the road of surgery option is always open.

### The treatment of spinal metastases from renal carcinoma: review of 108 cases treated from 1996 to 2011

S. Colangeli*[1], A. Gasbarrini[1], S. Bandiera[1], G. Barbanti Brodano[1], S. Terzi[1], L. Boriani[1], A. Corghi[1], R. Ghermandi[1], L. Babbi[1], M. Cappuccio[2], S. Boriani[1]

[1]Istituto Ortopedico Rizzoli (Bologna, IT);
[2]Ospedale Maggiore (Bologna, IT)

**Introduction** The skeleton is the third cancer metastasis most common site after lung and liver. Vertebral metastases for anatomical reasons probably are the most common bone site involved. The most frequent skeletal metastatic lesions are from the prostate, breast, kidney, lung and thyroid carcinoma. The vertebral metastasis may not only result in deterioration of quality of life, but also directly or indirectly cause the death of the patient. The skeletal metastases need multidisciplinary treatment in order to achieve the best possible local control of the lesion. The surgical indications for spinal metastasis are: intractable pain, neurological deficits (caused by compression of the myelo-radicular structures by the tumor mass or pathologic fracture of the vertebra) and the instability of the spinal cord, that causes an increasing mechanical pain and/or neurological deficit.

**Materials and methods** We analyzed 433 patients surgically treated for spinal metastases from 1996 to 2011. 108 cases of metastatic renal cell carcinoma were considered. 8 patients (7%) showed cervical spine localization, 40 in chest (37%) and 60 in lumbar spine (56%). 84 patients (78%) underwent embolization to reduce intraoperative blood loss. All patients were treated according to the Gasbarrini–Boriani algorithm for spinal metastases. The surgical treatments were schematically divided into: palliative, Debulking, En-bloc.

**Results** 50 patients underwent palliative treatments, 30 debulking and 28 en-bloc resections. 57 patients (53%) out of 108 died at a mean follow-up period of 21 months (min. 1–max. 95). Complications were further divided into 13 major (mechanical failures, high intraoperative bleeding) and 17 minor (wound dehiscence).

**Discussion** We decided to select patients with metastatic renal cell carcinoma because in our series they represent 25% of all spinal metastases, followed by indeterminate carcinoma (21%), lung cancer (17%) and breast cancer (13%). In about 85% of patients it was possible to restore or maintain movement, sensitivity, pain control with reduction of analgesic therapies. There are currently no specific works in literature dealing with treatment of spinal metastases from renal cell carcinoma to compare the data from this study.

**Conclusions** The aim of this paper is to give more information about this pathology, by using a homogenous series for Diagnosis, Site and Treatment.

### Percutaneous vertebroplasty and ablation for the treatment of vertebral metastasis: our experience

A. Zachos*, C. Meloni, F. Muresu, A. Ruggiu, C. Doria, P. Tranquilli Leali

Clinica Ortopedica, Università degli Studi di Sassari (Sassari, IT)

**Introduction** Percutaneous vertebroplasty (PVP) is a minimally invasive technique consisting of injection (PMMA) inside the weakened or collapsed vertebral bodies. It was performed for the first time by Galibert and Deramond in a case of symptomatic vertebral hemangioma and it is now used for the treatment of different kinds of vertebral collapse (osteoporosis, osteolytic bone metastases).

**Materials and methods** Percutaneous mini-invasive surgery (reduced blood loss) associates the ablation to the vertebroplasty for the treatment of solitary metastasis (vertebrae body). In our clinic, ablation is almost always associated with vertebroplasty. In our clinic, in the last 5 years, 76 patients (94 vertebrae), aged between 42 and 88 years (mean 65 years) were treated with vertebroplasty and ablation.

**Results** The postoperative elapsed were regular, with early mobilization and regression of pain. Periodic medical checks have been applied (1 month, 3 months, 6 months and then yearly) and X-ray with thoracolumbar radiological standards (1 month, 6 months and then yearly) that showed no problems of any kind at the level treated.

**Discussion** The effectiveness of PVP is now well described in literature, especially in the management of patients with osteoporotic and cancerous vertebral collapse.
**Conclusions** We believe that this minimally invasive technique is certainly a viable alternative to ''open'' traditional spine surgery.

### *C03—MUSCULOSKELETAL TUMORS AND METABOLIC DISEASES 3*

### Mini-invasive stabilization in patients with plasmocitoma of the spine

A. Corghi*, S. Terzi, S. Bandiera, G. Barbanti Brodano, L. Boriani, S. Colangeli, A. Gasbarrini, L. Babbi, R. Ghermandi, S. Boriani

Istituto Ortopedico Rizzoli (Bologna, IT)

**Introduction** Plasmocitoma, Multiple Myeloma (MM), is an oncoemathological diseases usually treated by chemiotherapy and/orradiotherapy. Spine Surgeon has a role in the diagnosis (biopsy CT guided) or in case pathological fracture (also when the fracture hasn't occurred yet but is very likely to happen) when he is asked for surgical stabilization.
**Materials and methods** We treated 23 patients affected by MM from May 2006 to February 2011: 13 patients by open surgery, 1 patient by vertebroplasty, 9 patients by mini-invasive stabilization.
**Results** Patients treated by mini-invasive surgery underwent shorter time of hospitalization (average 3 days) compared to those treated by open conventional stabilization (average 7 days). Moreover, patients treated by mini-invasive surgery could undergo adjuvant radiotherapy more quickly compared to those treated by open surgery.
**Discussion** Mini-invasive technique is useful in pathological fractures type A (according to Magerl classification) and when the risk for pathological fracture is high. Advantages of this technique is the reduction of the risk of neurological damage due to the fracture itself and the reduction of the pain (these results are achieved also with an open procedure); moreover it allows the patients to underwent Chemiotherapy and /or radiotherapy in a shorter time, without delays due to problems with the surgical wound or other complications requiring longer periods of hospitalization.
**Conclusions** When indication occurs, mini-invasive surgery in patients with MM of the spine is to be chosen because it's characterised by less postoperative complications, shorter hospitalization, and rapid application of chemiotherapy and/or radiotherapy.

### The use of demineralized bone matrix and mesenchymal stem cells concentrated for the treatment of the simple bone cysts

L. Cevolani*, D.M. Donati, T. Frisoni, B. Dozza, E. Lucarelli, S. Giannini

Istituto Ortopedico Rizzoli (Bologna, IT)

**Introduction** The simple bone cysts are benign lesions that are generally located in the epiphysis of the long bones during growth. The most frequent locations are the omerus and the femur. Cysts are often asymptomatic and frequently healed when skeletal maturity is reached. However, the high risk of pathological fractures justifies the treatment. The aim of the study is to develop a new mini-invasive technique that can reduce the number of hospitalizations.
**Materials and methods** The technique consists of a single injection with bone marrow (concentrated using the Cascade® method) and demineralized bone matrix. From November 2007 to February 2011 we treated 62 patients aged 4 to 25 years (average 10). The mean follow-up was 24 months (6–40 months). The patients were evaluated clinically and radiographically 2, 6 and 24 months after the operation and then every 6 or 12 months. The treatment was evaluated using the radiographic classification by Neer.
**Results** There was a 77% success in treatment. The 16% of the cases needed more than one treatment and the 13% of the cases had a pathological fracture. After 24 months from the operation, Neer's index I or II was presented by 68% of the patients, Neer III by 26% and Neer IV by 6%.
**Discussion** At present, there is not a final treatment for this pathology. The invasive treatment includes the curettage and the application of bone grafts, the multiple perforation of the cystic cavity or the application of cannulated screws of titan or of hydroxyapatite. These techniques are very invasive; the curettage can also cause a growth place damage. Less invasive procedures are the injection with steroids or with bone marrow with or without adjuvant. However, these techniques require multiple hospitalizations and are poorly tolerated by the patients. Our technique allows obtaining very satisfactory results with a single hospitalization.
**Conclusions** Our treatment is efficacious for the therapy of simple bone cysts. This technique can reduce the cost of disease management because of the lows number of hospitalizations needed. This technique cannot reduce the fracture risk.

### 100 prosthesis for bone tumors at the Department of Oncological Orthopaedics of Regina Elena National Cancer Institute: preliminary results

N. Salducca*, C. Zoccali, L. Favale, U. Prencipe, F. Erba, G. Teori, R. Biagini

Divisione di Ortopedia Oncologica, Istituto Nazionale Tumori Regina Elena (Rome, IT)

**Introduction** In the last years, because of the improvement of chemotherapy, the improvement of prognosis of several primitive and secondary tumors and the increase in accuracy of radiological technique allowing an early diagnosis, there has been an increase of resections and prosthesis reconstructions; moreover the increase of survival has caused the onset of biomechanical long-term complications.
**Materials and methods** During the period between February 2005 and 2011, one hundred patients were treated with tumoral resection and prosthesis reconstruction. We report a little prevalence of females (51%); the site most involved was the femur (52%). The diagnosis more frequent was osteosarcoma (22%) and metastasis (50%), followed by chondrosarcoma (12%), giant cell tumor (4%) and Ewing Sarcoma (3%).
**Results** These 102 cases are 79 femoral reconstruction (53 proximal femur, 20 distal femur, 3 total femur, 3 diaphiseal femur), 9 tibial reconstructions, 2 double reconstructions (distal femur and proximal tibia), 12 humeral prosthesis (8 proximal humerus, 1 total humerus, 3 elbow). Functional results, scored with MSTS system, were good or excellent in most of cases. Major complications were infections (7% of cases) and mechanical failure (7% of cases) as dislocations, breaking or mobilization of the prosthesis. We reported 5 local relapses, in 4 patient skeletal metastasis and in 2 lung metastasis.

**Conclusions** Surgery of primitive or isolated metastatic lesions of musculoskeletal apparatus is more frequent. The possibility to perform complex reconstruction allows to guarantee a better life quality than in the past. The improvement of the re-insertion tendons technique on the prosthesis should allow a further functional improvement.

### Imatinib Mesylate (IM-GLIVEC) activity in a patient with recurrent pigmented villonodular synovitis/tenosynovial giant cell tumour (PVNS/TGCT). A case report from the Regina Elena National Cancer Institute (Rome)

V. Ferraresi*[1], C. Zoccali[2], C. Nuzzo[1], U. Prencipe[2], M. Tedeschi[1], G. Teori[2], R. Sciuto[3], R. Biagini[2]

[1]Divisione di Oncologia Medica A, Istituto Nazionale Tumori Regina Elena (Rome, IT);
[2]Divisione di Ortopedia Oncologica, Istituto Nazionale Tumori Regina Elena (Rome, IT);
[3]Divisione di Medicina Nucleare, Istituto Nazionale Tumori Regina Elena (Rome, IT)

**Introduction** PVNS/TGCT is a rare pathological entity affecting the synovium. Surgery is the treatment of choice but relapses may frequently occur and re-excision is sometimes troublesome. Recently, this disease has been recognized as a benign neoplastic process with a specific translocation involving the collagen 6A3 gene and the M-CSF gene. This fusion gene encodes for a fusion protein which attracts M-CSF receptor expressing cells (macrophages and monocytes). IM has recently been reported to block M-CSFR activation so disrupting the paracrine loop found responsible for PVNS/TGCT growth.

**Materials and methods** We describe the clinical outcome of a 47-year-old male affected by recurrent PVNS of left knee and treated with IM. In May 2007 the patient underwent arthroscopy with excisional biopsy of a nodulation of the fibro-cartilage of the left knee with histological diagnosis of PVNS. In October 2007 and April 2008 the patient was submitted to excision of multiple relapses of disease located in the popliteal recess. Due to the new appearance of multiple relapses judged not more amenable of surgery, the patient was treated with systemic steroids without any clinical benefit. The patient came to the observation of our orthopedic department in September 2009 and surgery, unless important functional sequaele, was excluded. A MRI performed in January 2010 showed multiple relapses of disease involving the posterior compartment of left knee and a soft swelling of cm 7 × 4.5 was palpable at clinical examination. The patient presented objective impairment and pain during left leg flexion. Based on case reports on IM in PVNS, an off label use of the drug was authorized by our Ethical Committee and from May 2010 until today the patient is being treated with IM at the dose of 400 mg p.o./day.

**Results** A metabolic FDG-PET response was observed at month 3 with a reduction of local max SUV from 8.5 to 5.5. The response was maintained at month 8. This finding was associated to the almost complete disappearance of the palpable mass at clinical examination. The patient reported a progressive improvement in left leg flexion and pain relief. The treatment is still ongoing with a good tolerability except for mild gastrointestinal toxicity and mild fluid retention.

**Conclusions** IM proved to induce a durable partial response in a patient affected by relapsing PVNS/TGCT even if the optimal duration of therapy still remain to be determined. The efficacy of others tyrosin-kinase inhibitors, such as nilotinib, in PVNS/TGCT is under investigation.

### *C04—MUSCULOSKELETAL TUMORS AND METABOLIC DISEASES 4*

### Risk of fragility fractures in osteopenic patients: sub-analysis of the INDACO2 survey

G. Iolascon*, A. Gimigliano, A. Tirelli, F. Gimigliano

Seconda Università degli Studi di Napoli (Naples, IT)

**Introduction** In Italy the costs of osteoporosis and of fragility fractures are estimated up to 2 billion Euros in a year. BMD is useful for the diagnosis of osteoporosis (diagnostic threshold) but is not sufficient to identify all the people at risk of fracture (therapeutic threshold). In fact, it is possible that also in the osteopenic population a fracture can occur. To identify these patients is important in order to reduce the clinical and social consequences of the fracture itself.

**Materials and methods** In the study INDACO2, an epidemiological survey promoted by the Italian Society of Orthopedics and Traumatology (SIOT), we collected data on 7,355 patients in 145 divisions of orthopedics and traumatology in a 6 months period. It was used a form collecting data not only on the presence of fragility fractures but also all the informations necessary to calculate the FRAX index. The therapeutic threshold for the FRAX HIP index is =3% and for the FRAX MAJ index is =20%.

Of the 7,355 forms collected we eliminated those incomplete and considered only patients with a T-Score between −2, 5 e − 1, so the final analysis was done on 472 patients.

**Results** Of the 472 patients evaluated (mean age 73.7 years), 201 (42.4%) had a fragility fracture: 92 a hip fracture, 97 a vertebral fracture and 33 a fracture in another fragility site. 69.15% of osteopenic patients without a fracture had a FRAX HIP score =3% and 22.88% a FRAX MAJ =20%. Instead of the 92 osteopenic patients with hip fracture, 85.87% had a FRAX HIP score =3%, and 63% had a FRAX MAJ =20%. Considering osteopenic patients with vertebral fractures, 71.16% had a FRAX HIP score =3 and 36% FRAX MAJ =20%. Among those with fragility fracture in other sites, 75.75% had a FRAX HIP score =3% and 39.4% a FRAX MAJ =20%.

**Discussion** In our osteopenic population the risk of sustaining a fracture within ten years is high both in people with or without prevalent fragility fractures. Probably, in our population, the increased risk of fragility fractures is mainly related to old age of patients.

**Conclusions** Osteopenia represents a risk factor for fragility fractures also in older people. It is important to treat osteopenic elderly patients, in presence of other risk factors, to prevent hip fracture.

### Frequency of hip fractures in osteopenic population: sub-analysis of the INDACO2 survey

G. Iolascon*, S. Gatto, M. Donnarumma, E. Theodoridou, A. Riccardo, G. Del Gaudio, F. Gimigliano

Seconda Università di Napoli (Naples, IT)

**Introduction** Osteopenia is a condition in which bone mineral density is lower than normal (T-score ranging from −1.0 to −2.5). However, we do not know whom of the osteopenic subjects will develop osteoporosis and fragility fractures, or what is the role of the calcium-vitamin supplementation and osteo-metabolic drugs in this population. The aim of our study was to evaluate the relationship between calcium and vitamin D intake and risk of hip fracture in the osteopenic population.

**Materials and methods** In an epidemiological survey organized by the Italian Society of Orthopedics and Traumatology (SIOT), 6,465 patients aged over 65 years were recruited, referring to 143 Orthopaedic/Traumatology divisions, widespread on Italy, over a 6 months period. Data were collected by administering a questionnaire, investigating several aspects of the patient's medical history including previous use of calcium and vitamin D supplementation and osteo-metabolic drugs.

**Results** Of the 1,941 patients for whom a T-score value had been reported, 472 (24.3%) were osteopenic (T-score ranging from −1.0 to −2.5). Of these 90 were taking a calcium and vitamin D supplementation, 100 were taking osteo-metabolic drugs, 89 were taking both calcium and vitamin D supplementation and osteo-metabolic drugs, and 191 were not treated for osteoporosis. Of 472 patients evaluated, 92 (19.5%) had a hip fracture: 16 of these (17.3%) taking calcium and vitamin D, 21 (22.8%) taking osteo-metabolic drugs, 12 (13%) taking calcium, vitamin D plus osteo-metabolic drugs and 43 (46.7%) not receiving any therapy for osteoporosis.

**Discussion** In our population, 19.5% of patients with T-score between −1 and −2.5 had a hip fracture and only 35.8% of these took osteo-metabolic drugs, 17.3 % took calcium and vitamin D supplementation and as much as 46.7% did not undertake any therapy for osteoporosis.

**Conclusions** In the population over 65 years with a hip fracture, osteopenia should be considered an important risk factor for hip fractures. In these subjects it is important to identify the presence of other possible risk factors and eventually recommend a pharmacologic approach with at least calcium and vitamin D supplementation.

### Prevalence of hip fractures in subjects with secondary osteoporosis: sub-analysis of the survey INDACO2

G. Iolascon*, S. Gatto, A. Capaldo, G. Di Pietro, M. D'Amico, F. Gimigliano

Seconda Università di Napoli (Naples, IT)

**Introduction** Several conditions fall under definition of secondary osteoporosis and can increase bone fragility and fracture risk. The objective of this study is to assess the presence of hip fractures in this population, particularly in subjects who chronically assumed glucocorticoids.

**Materials and methods** On behalf of the Italian Society of Orthopaedics and Traumatology (SIOT) between January–June 2010 we conducted an epidemiological survey (study INDACO 2) on 6465 subjects aged over 65 years, recruited in 143 Orthopedic/Traumatologic Centers, widespread on Italy. In each Center 30 patients with hip fracture and 30 age-matched controls were recruited. Subjects were administered a questionnaire investigating, among other fields, the possible diagnosis of secondary osteoporosis and possible chronic treatment with glucocorticoids.

**Results** Of the 6,465 patients enrolled, 5,700 had a diagnosis of osteoporosis: 4,365 (76.58%) had a diagnosis of primary osteoporosis and 1,335 (23.42%) of secondary osteoporosis. Of the 4,365 patients with primary osteoporosis, 2,290 (52.46%) had a hip fracture and 1,335 with a diagnosis of secondary osteoporosis, 576 (43.14%) had a hip fracture. Patients with osteoporosis secondary to chronic treatment with glucocorticoids were 592 (44.34%) and of these 219 (36.99%) were fractured.

**Discussion** Our study confirmed the high prevalence of secondary osteoporosis in our population. Chronic treatment with glucocorticoids is one of the main causes of secondary osteoporosis accounting for over one third of cases. In our population (mean age 76.9 years) the 36.99% had a hip fracture. Generally, in our data, hip fracture is less frequent in the population affected by secondary osteoporosis (43.14%) compared with population with involutive osteoporosis (52.46%).

**Conclusions** Chronic treatment with glucocorticoids is the most frequent cause of secondary osteoporosis. For Italian Regulatory Agency for Drugs, it's refundable only the therapy for glucocorticoid-induced osteoporosis (GIOP). Nevertheless both diagnosis and treatment of GIOP are however underestimated.

### Critical analysis on lower extremity amputations (LEAS) in the diabetic patients

M. Rosati*[1], A. Baluganti[1], N. Piolanti[1], A. Terzuoli[1], A. Piaggesi[2], L. Rizzo[2]

[1]I Clinica Ortopedica, Università di Pisa (Pisa, IT);
[2]Sezione di Diabetologia e Malattie del Metabolismo, Dipartimento di Endocrinologia e Malattie del Metabolismo (Pisa, IT)

**Introduction** It is well established that the management of diabetic foot ulcers performed by a multidisciplinary team, consisting of a surgeon, an anesthetiologist and a diabetologist, have a better outcome and decreases the incidence of major amputations. We also think that the diabetic patient who has undergone a major amputation should be treated by a multidisciplinary team in order to reduce mortality, morbidity and time to wound healing.

**Materials and methods** At the I Orthopedic Department, University of Pisa, from October 2007 to January 2010 over 70 major lower extremity amputation due to vascular gangrene, diabetic ischemia, burns and cancer of the lower limbs were carried out. In this paper we focus our attention on the 51 major amputation performed on 48 diabetic patient. The patients were divided into two groups according to if they were referred by the diabetology department, which admitted them pre-operatively (group A), or if they were referred by other departments of our hospital (group B).

**Results** 30 patients [age 70.7 ± 9.5 years, duration of diabetes (DD) 22.9 ± 11.3 years, HbA1c 8.4 ± 1.6%] in group A and 18 patients [age 73.4 ± 11.0 years, DD 24.1 ± 11.1 years, HbA1c 8.8 ± 1.4%] in group B. Group A patients had comorbidities in 88% of cases vs. 67% in group B (chi-square 12.645, $p = 0.0006$). In group A 70% of patients underwent a leg amputation and 30% a thigh one, while in group B 28% underwent leg amputation and 72% a thigh one (chi-square 35.294, $p < 0.0001$); 64% of patients in group A vs. 44% in group B healed after LEA (chi-square 8.052, $p = 0.0069$); healing time was 76.7 ± 42.7 days in group A vs. 272.5 ± 121.7 days in group B ($p < 0.01$). At follow-up 33% of patients in group A vs. 65% in group B were dead (chi-square 20.854, $p < 0.0001$); 33% in group A were ambulatory with prosthesis vs. 11% in group B (chi-square 14.103, $p = 0.0003$).

**Discussion** The ischemia-related chronic peripheral vascular insufficiency is the most common cause of major amputations in the lower limbs. Accurate surgical techniques, performed by experienced surgeons, can improve the survival rates. Nevertheless, the mortality and complications following major amputation remain high.

**Conclusions** Despite their higher prevalence of comorbidities, the patients referred for LEA from the Diabetological Department and managed with an integrated multidisciplinary strategy, had a better outcome of the surgical procedure, a shorter healing time, a higher survival rate and a better functional outcome.

### Efficacy of the anabolic therapies in severe osteoporosis: experience of a team of endocrinologists and orthopaedic surgeons and clinical results

E. Pola*, D. Colangelo, A. Fusco, M. Mormando, G. Di Giacomo, B. Rossi, L. De Marinis, C.A. Logroscino

Università Cattolica del Sacro Cuore, Policlinico “A. Gemelli” (Rome, IT)

**Introduction** Anabolic therapies represent a major advance in the management of severe osteoporosis, and they may provide significant benefit to those patients with post-menopausal osteoporosis in whom antiresorptive therapy has proven insufficient. Parathyroid hormone (PTH) and human recombinant PTH peptide 1–34 (Teriparatide) have demonstrated an increase in bone mineral density (BMD) and a significant reduction of risk vertebral fractures in patients with osteoporosis when given for 18–24 months.
**Materials and methods** We retrospectively analyzed the safety, efficacy and compliance to therapy with anabolic agents in 53 patients (M/F: 7/46) with severe primary osteoporosis resistant to antiresorptive therapy. Patients were followed at an Outpatient Centre of Osteoporosis managed by a team of orthopaedic surgeons and endocrinologists from 2007 to 2010. Thirty patients underwent percutaneous kyphoplasty and 23 were treated with brace before starting the anabolic therapy. The anabolic agent was administered as a daily 20 μg subcutaneous injection, and supplementation of vitamin D was given, when necessary. The change in BMD value was measured at the beginning of therapy and after 18 months through lumbar and femoral DEXA scan.
**Results** A total of 50 patients completed the 18 months treatment with Teriparatide. Three patients (5.35%) discontinued the treatment due to side effects occurring during the first 3 months of therapy (hypercalcemia occurred in 2 patients, and nausea in 1). Before treatment, all patients had vertebral fractures and suffered from chronic and/or acute lumbar pain. After the beginning of the anabolic therapy, 47% of patients showed a significant improvement of lumbar pain. At the end of the 18 months of treatment, there was a substantial increase in BMD at both lumbar spine (+9%) and femoral neck (+5%).
**Discussion** Differently from bisphosphonates, which reduce bone turnover through the inhibition of osteoclastic activity, the intermittent administration of PTH stimulates osteoblastic function, increases trabecular bone mass and improves architecture of both trabecular and cortical bone. Moreover, PTH reduces the lumbar pain in a significant percentage of patients with multiple vertebral fractures; this additional analgesic effect improves quality of life of elderly patients, often affected from other co-morbidities.
**Conclusions** Anabolic therapy is safe and effective in the treatment of severe osteoporosis. Adherence to therapy is very high, despite the daily administration. Anabolic agents could be particularly effective in patients with vertebral collapses from glucocorticoids-induced osteoporosis and even in male osteoporotic patients.

### Multiple vertebral fractures in an adult male suffering from secondary osteoporosis: still an underestimated reality

R. Cosso*[1], G. Galli[1], P. Bungaro[1], M. Perozzi[1], A. Falchetti[2]

[1]Casa di Cura “Villa Erbosa” (Bologna, IT);
[2]Dipartimento di Medicina Interna Università di Firenze (Florence, IT)

**Introduction** Male osteoporosis (MOP) is an important social problem, amplified by the increasing life expectancy. Fifty percent of cases of MOP are secondary to medications (glucocorticoids) and/or diseases (hypogonadism and other endocrine disorders) and alcohol abuse. MOP is still underdiagnosed and undertreated, often revealing only after the occurrence of a fragility fracture. We present the case of a 58 year old male with a spontaneous fracture of L2 at the age of 54 years.
**Materials and methods** The patient presented us with a spine radiograph showing the outcome of atraumatic fractures at T5, T6, T7, as well as L2, and a DXA scan revealing a lumbar bone mineral density (BMD) average of −1.8 SD, with values of −2.9 SD and 3.4 SD at L1 and L2, respectively, and a femoral osteopenia. The family history for risk factors for OP/fractures was positive for ankylosing spondylitis, diabetes mellitus type 2, kidney stones and hypercholesterolemia. Personal history revealed an important periodontal disease with spontaneous loss of 5 teeth in last 6–7 years, daily alcohol consumption above the general average, diffuse spondylosis, low consumption of milk dairy products. At visit we reported a body mass index of 30.2 with android obesity and mild muscle atrophy. Therefore, we suggested to assess bone turnover and hormonal status.
**Results** These surveys revealed a picture of hypovitaminosis D and primary hypogonadism. The patient was put under treatment with alendronate 70 mg, 1 tablet a week, and cholecalciferol 300,000 IU vials, 1 vial orally repeated after 2 weeks and then calcium carbonate and cholecalciferol, orodispersive tablets, 1.2 grams/day + 800 IU/day. Then, we request an endocrinological evaluation for hypogonadism and likely plurimetabolic syndrome, in which low levels of circulating testosterone and vitamin D production may coexist.
**Discussion** This is a condition of MOP secondary to hypogonadism and vitamin D deficiency in the presence of risk factors for osteoporosis/fragility fractures, either in family or personal history (low calcium in diet and alcohol abuse).
**Conclusions** Although androgens can prevent the loss of cancellous bone and stimulate the sub-periosteal apposition of cortical bone, the anabolic effect of testosterone on bone and muscle, is limited by the high incidence of androgenic side effects. Hypogonadism is the only situation in which the benefits of using formulations of testosterone may outweigh their side effects.

### *C05—TRAUMATOLOGY 1*

### Indications and limits of the Fixator TGF “Gex-Fix” in fractures of the proximal end of the humerus

N. Galvano*, A. Parlato, A. D’Arienzo, M. D’Arienzo

[1]Clinica Ortopedica, AOUP Policlinico (Palermo, IT)

**Introduction** In the last years we observed an exponential increase of the incidence of fractures of the humerus, caused by road trauma and osteoporosis. Treatment is conservative in 75% of cases, in the remaining cases is surgical. Classifications found in literature are the morphological of Neer; biological of AO/ASIF and descriptive of Hertel, the most used, characterized by the system LEGO with four bricks: head, shaft, small and great tuberosity. The types of treatment include the conservative one with Desault’s bandage, prosthetic replacement, and synthesis through various means, including TGF (tension guides fixator) GexFix. The technique involves the introduction of Steinman pin to keep manual reduction, the introduction of two K-wires on the humeral head, the removal of the Steinman pin, and the introduction of two drilling fiches on the shaft. On the wires and on the chips are mounted hubs connectors, connecting the outer bar and tensioning system.

**Materials and methods** Our experience from December 2007 to January 2011, includes 64 proximal end humeral fractures (66% at 2 fragments) treated with Fixator TGF, minimum age 42 years and maximum 84. The post-operative recovery is early, the commuters movements are granted 24 h after treatment, the passive after 48 h, the active assisted are less painful than other surgical techniques. The removal takes place in six weeks.
**Results** The results, evaluated with VAS score were good in 82% of cases. This technique has less risk of infection, bleeding and surgical time than other surgical techniques but it has increased risk of cephalic necrosis.
**Discussion** Our results, after two and a half years of experience confirm that the best indication of this fixator is reserved for fractures with one, two or three fragments also on osteoporotic bone, in patients with moderate functional demands, given the opportunity to allow early active mobilization with the commuter movements and passive movements. The limits of this fixator were evident in fractures in which closed reduction is not possible and in fracture with 3–4 fragments displaced in varus because the Fixator TGF has less stability than other systems such as plate or cage.
**Conclusions** The short learning curve, reduced surgical time and risk and low cost encourage to use this technique, an increase of the series and follow-up will confirm what has been alleged. It is important to avoid failures, to use this Fixator in the cases with a specific indication.

### Pinnig as an alternative treatment for proximal humerus fractures

G. Manetti, L. De Luca*, M. Sangiovanni, M. Cardini, U. Rossi

SOD Ortopedia Traumatologica, A.O.U. Careggi (Florence, IT)

**Introduction** The treatment of proximal humerus fractures is still controversial. These represent 20% of fractures in this region. Their treatment, especially in elderly patients with osteopenia, is still debated.
**Materials and methods** We treated 54 patients (36 women, 18 men) between 2003 and 2010, with the technique of percutaneous pinning with threaded pin. In 10 cases it was necessary to make a mini-open access with deltoid splitting to achieve reduction of the fracture. The average age of patients was 65 years (range 60–82). We treated 30 fractures of type 2 according to the Neer classification, 21 type 3 and 3 type 4. In 46% of cases had involved the dominant limb. Complications were evaluated intra-and post-operative. We evaluated the clinical results with the Neer Score and clinical follow-up with X-ray ROM and VAS pain scale.
**Results** We had no intraoperative complications. The pins were removed between 3 and 8 weeks post-surgery. The results were excellent in 27.7% of cases, good in 38.8%, mild in 22.3 and 11.2% was bad. The average ROM of the shoulder operation was 113.2° of anterior elevation, external rotation 41.4°, 62.3° of internal rotation and 103.1° of abduction. We recorded three cases of infection with the pin in 2 cases that required early removal of the same, loosening of the pins in 4 cases (including 2 patients underwent a new synthesis) and avascular necrosis in 2 cases.
**Discussion** This technique involves an advantage in terms of reduced surgical time and bleeding, a reduction in the risk of avascular and aseptic necrosis, as we have less risk of injury to the humeral head blood supply. The disadvantages lie in the most approximate reduction in possible injury to anatomical structures and possible migration of the pin. We can also easily have a secondary loss of fracture reduction, as well as a greater risk of infection. Finally, patients treated with this technique require more frequent post-operative follow-up.
**Conclusions** This method of treatment is a viable surgical option for fractures at 2, 3 and 4 fragments in valgus. It looks like a solution that is not invasive, quick, but not easy. It is a good balance in elderly patients with co-morbidity.

### The treatment of proximal humeral fractures with antegrade locked intramedullary nail

E. Siuni*, M. Planta, F. Orgiano, I. Pusceddu, B. Orrù

Servizio di Chirurgia della Spalla SCOMR, Ospedale Marino (Cagliari, IT)

**Introduction** This study describes our experience with antegrade short locked intramedullary nail in the treatment of humeral fractures of the surgical neck and in the ones with extension of the line's fracture in the epiphyseal region. The anterograde closed nails are inserted through a small incision with minimal soft-tissue trauma and with respect to the periosteal vascularization. Possible damage of the rotator cuff has been evaluated with ultrasound studies after the surgery.
**Materials and methods** From June 2008 to September 2010, 31 patients (18 females and 13 males; range, 25–84 years) were treated by surgical internal fixation with a short locked intramedullary nail. The mean follow-up was 22 months (range, 3–33) and all patients were evaluated with clinical, radiographic and ultrasound examination.
**Results** The mean Constant score was 80 (min. 72, max. 95). The fractures healed in an average time of 2,5 months. The most functional outcomes were obtained by patients with two-part fractures of the surgical neck, but in the ones of epiphyseal region the result depended on a good quality of bone fragments' reduction during the surgery. Among the complications, 1 patient underwent surgical revision for humeral fracture in which the short locked intramedullary nail was removed and a long one was inserted, another patient had a delayed union of the surgical neck. In the remaining cases the results have been good and no nails have been removed yet. The rotator cuff has not shown significant damages with regard to the surgical procedure.
**Discussion** Surgical treatment of proximal humeral fractures with antegrade locked intramedullary nails allows, with a meticulous intraoperative management, a stable fixation and thus, an early mobilization with strict postoperative rehabilitation of the shoulder and better outcomes.
**Conclusions** The results obtained with the short intramedullary nail indicate that it represent an efficacious and reliable therapeutic solution in particular for surgical neck fractures because of its minimal invasive surgery (close reduction and internal fixation).

### Proximal humeral fracture treatment with locking plate and polyaxial screws

L. Murena*[1], S. Turino[1], S.V. Boscarino[1], F.A. Grassi[2], P. Cherubino[1]

[1]Dipartimento di Scienze Chirurgiche Ricostruttive e Tecnologie Avanzate, Università degli Studi dell'Insubria (Varese, IT);
[2]S.C. di Ortopedia e Traumatologia, Azienda Ospedaliero-Universitaria "Maggiore della Carità", Università degli Studi del Piemonte Orientale "A. Avogadro" (Novara, IT)

**Introduction** The use of locking plates in the treatment of proximal humeral fractures is constantly increasing. Some of the available locking plates are implemented with polyaxial screws system, which should be more suitable for the different fracture patterns. In this study we analyzed the results obtained with this type of implant.

**Materials and methods** We reviewed retrospectively all the patients that were treated for a proximal humeral fracture through an open reduction and internal fixation with a locking plate with polyaxial screws in our department between July 2006 and July 2009. We evaluated 41 patients, 25 women and 16 men (mean age 55.43 years). The shortest follow-up was 12 months, the longest 50 months (mean follow-up was 28 months). We assessed the clinical outcome (ROM, VAS, Constant Score and Oxford Shoulder Score) and the radiological outcome (true A-P view, axillary view and lateral Y-view of scapula).

**Results** We evaluated during the follow-up 39 patients. The mean range of motion parameters were: elevation 153.18°, abduction 148,93° and external rotation 52.12°; the mean results for the clinical scores were: VAS 2.03; Constant Score 76.09; OSS 16.28. In 36 patients we observed radiological healing of the fracture. In the remaining cases we observed: 1 non-union (2.5%); 1 plate mobilization from the diaphiseal part (2.5%); 1 post-traumatic necrosis of the proximal epiphisis (2.5%). Further, we observed in 1 patient (2.5%) an epiphyseal screw protrusion in the articular space.

**Discussion** The main purpose of surgery in proximal humeral fractures is to achieve a sufficiently stable osteosynthesis to allow early articular mobilization. The results found in the present study are comparable with the data reported in literature: the outcomes observed with polyaxial and with monoaxial screws systems are quite comparable. In particular the incidence of complications was not increased, due to the multi—directional locking system. This feature makes the plate more suitable to the different fracture patterns and could make easier the implant.

**Conclusions** The locking plate with polyaxial screws represents a valid surgical option in the treatment of proximal humeral fractures. Anyway it is essential a meticulous surgical technique to minimize the incidence of complications and to achieve good results.

### Fixation of proximal humerus fractures with Polarus® intramedullary nail

M. Leigheb*, G. De Falco, R. Ceffa, F.A. Grassi

AOU Maggiore D.C., S.C. Ortopedia-Traumatologia (Novara, IT)

**Introduction** Surgical treatment of proximal humeral fractures is influenced by several factors, both local and systemic. Intramedullary nailing is a surgical option that has been increasingly adopted by surgeons during the last years. Aim of the study is to evaluate the results achieved in a consecutive series of patients treated with the Polarus® locking humeral rod.

**Materials and methods** Forty patients (29 women, 11 men) with a mean age of 70.4 years (range 40–94), all treated with the Polarus® rod between 2008 and 2009 at a single institution, were included in this study. According to Neer classification, there were 36 two-part fractures, 2 three-part fractures and 2 two-part fracture-dislocations; in 2 cases the fracture rim was extended from the surgical neck to the humeral diaphysis (11-A2 + 12-A1 according to the AO classification). After the operation, the shoulder was immediately mobilized for personal hygiene, but the rehabilitation protocol was individualized according to the reliability of fixation. The clinical-functional outcome was evaluated using the Absolute Constant Score (CS) and the Relative Constant Score (CS-R), the latter being the score ratio with the CS of the opposite shoulder. X-rays were taken at 1, 2, 3 and 6 months.

**Results** All the patients were followed-up for a minimum of 6 months (range 6–32). Fracture healing was achieved in 39 patients. Five (12.5%) late complications were observed: 1 surgical neck nonunion, 1 avascular necrosis of the humeral head, 1 malunion with intrarticular protrusion of a proximal screw and subacromial impingement, 1 distal screw mobilization and 1 periosteal osteolysis of unknown origin. The mean CS and CR-R scores were respectively 69.9 (range 25–96) and 81.1% (range 30.1–100). Pain was absent in 27 patients (67.5%), mild in 11 (27.5%) and moderate in 2 (5%). Active ROM was excellent or good in 32 patients (80%), fair in 6 (15%) and poor in 2 (5%). Better functional results were observed in patients under 70 years and in those who started active shoulder motion earlier (before 2 weeks post-op).

**Conclusions** The Polarus® humeral nail showed effectiveness and reliability for the fixation of two-part fractures (surgical neck). In most of the cases it allowed early shoulder mobilization, which is essential for achieving a satisfactory functional outcome. Radiographic monitoring for a minimum of 3 months after the operation is necessary in order to detect potential complications.

### Percutaneous fixation of proximal humerus fractures using a new fixation system

G. Logroscino*, G. Milano, F. Donati, L. Deriu, C. Fabbriciani

Istituto di Clinica Ortopedica, Università Cattolica del Sacro Cuore (Rome, IT)

**Introduction** Percutaneous fixation of proximal humerus fractures is a widely accepted technique since the risk of surgery-related avascular necrosis is low. The purpose of the present study was to describe a new surgical technique, using a new fixation system, and to show its clinical and radiological results in a cohort of patient with proximal humerus fracture.

**Materials and methods** Twenty patients with a proximal humerus fracture underwent reduction and internal fixation with MIROS percutaneous fixation system. Twelve patients had a two-part fracture (surgical neck), the others (8 cases) a three-part fracture. Comminuted and not-reducible fractures were excluded from the study. Wires were removed after 3–4 weeks. Every patient was evaluated clinically (Constant Score, VAS pain, SF-12) and radiologically (AP and axillary views of the affected shoulder) at 1–2–3–6 months after surgery. Surgical Technique Closed reduction was obtained under fluoroscopic control. Circumflex nerve course was drawn on the skin. Two threaded retrograde wires were used to fix the fracture. Once a stable reduction between epiphysis and diaphysis was obtained, two anterograde intramedullary wires (2.5 mm. wires, MIROS system) were placed down to distal metaphysis achieving compression and ultimate stability. Wires were then fastened to MIROS clips, in order to obtain 3D stability thus preserving high elasticity of the construct.

**Results** Radiological evaluation showed that reduction was maintained after 3–4 weeks, therefore wires were removed (19 cases). Bone healing occurred after 8–12 weeks. In one case, reduction failed and open reduction was performed. At 6 months follow-up (min. 2 months, max. 1 year) mean scores recorded were: Constant 82/100, SF-12 (PCS 45.2; MCS 55.3), VAS 12 mm.

**Discussion** Percutaneous fixation of proximal humerus fracture was considered an alternative technique in proximal fractures treatment, but many limitations were reported in the past, in particular in old osteoporotic patients. In this study percutaneous fixation with wires showed to be a safe and reliable technique for two and three-parts unstable fractures of the proximal humerus. This new system allows to obtain excellent results if used with proper indications (2–3 parts, non comminuted fractures), reducible with closed reduction.
**Conclusions** Low invasiveness and relative safety of this technique permit to significantly widen indications. This is extremely important in the old osteoporotics patients in which good results can be obtained avoiding open reduction and internal fixation (ORIF). However, a larger cohort of patients and a longer follow-up is necessary to confirm this preliminary results.

### Complex pluri-fragmentary fractures of the distal humerus: our experience with double-plate

L. Nalbone*, M. Ferruzza, S. Mannino, A. Parlato, M. D'Arienzo

Clinica Ortopedica e Traumatologica, Policlinico Universitario "Paolo Giaccone" (Palermo, IT)

**Introduction** The serious pluri-fragmentation with bone loss in fractures of the distal humerus, especially in individuals with osteoporosis, often gives unsatisfactory results and puts to the test the ability and experience of the orthopaedic surgeon because insufficient stability can lead to the discontinuity of the fragments especially if early mobilization is begun while the lengthening of immobilization time leads to rigidity.
**Materials and methods** To reduce the above-mentioned problems, between 2004–2009, we treated 15 patients with complex displaced pluri-fragmentary fractures of the humerus using a synthesis with double plates to assure considerable stability. The patients were from 25 to 64 years old (average 38.4). The follow-up ranges from 18 months to 5 years. Functional re-education was begun at an early stage.
**Results** In the cases under control, 75–85% had good and excellent outcomes and were maintained for a long time with a good recovery of functional activity. The range of joint movement was on average about 122° with an average pronation and supination of about 75°. There were no post-operative infections or dislocations.
**Discussion** The plury-fragmentary, displaced complex fractures of the distal humerus should be considered a challenge to the expert surgeon. Various means of synthesis are used, but the double-plates one is the one that allows a more accurate anatomical reconstruction and better stability especially in elderly patients with reduced bone density.
**Conclusions** Internal fixation of joint fractures of the distal humerus with a double plate is a valid procedure with good functional results deriving from the complete stability and early rehabilitation.

### Transdeltoid approach for plate osteosynthesis with angular stability in proximal humeral fractures

A. Rota, P. De Santis*

Ospedale S. Pertini (Rome, IT)

**Introduction** Fractures of the proximal humerus are common (about 4–5% of all fractures) and increasing because of their association with osteoporosis. Treatment options include the use of the proximal nails, plates, percutaneous or minimally invasive techniques; the incidence of complications in the surgical treatment, reported in different series, is between 11 and 50%. The open reduction and internal fixation (ORIF) of proximal humeral fractures with conventional plate has been associated with loss of reduction, mobilization of the screws and osteonecrosis; in recent years have been developed plates with angular stability to keep anatomical reduction with a strong fixation, especially in osteoporotic bone.
**Materials and methods** From June 2009 to January 2011, in the Orthopedics and Traumatology Department of S. Pertini Hospital (Rome), 32 patients with proximal humeral fractures (11-A, 11 B1-2, 11C1-2 AO classification) were surgically treated performing a plate osteosynthesis with angular stability through the transdeltoid approach, with isolation of the circumflex nerve. In this study fractures 11-B3 and 11-C3 are not included, because the glenohumeral joint dislocation is a contraindication to the use of this approach in osteosynthesis of proximal humeral fractures.
**Results** In all cases treated by transdeltoid approach has been isolated circumflex nerve and has been possible always reduce indirectly anatomical and surgical neck of humerus. In this study, although with short follow-up, there was the consolidation in 92% of cases, no lesion of the circumflex nerve and no avascular necrosis (AVN) of humeral head.
**Discussion** The poor results and high complication rate (nonunion or AVN of the humeral head) after proximal humerus osteosynthesis by deltopectoral approach may be due to devascularization of fragments, during the massive soft tissue dissection, and reduction of blood supply to the humeral head.
**Conclusions** Transdeltoid approach allows the preservation of the vascularization of the proximal humerus, provides a direct approach to the humeral tuberosity, also giving the opportunity to reduce indirectly anatomical and surgical neck; when using this approach is always recommended to isolate the circumflex nerve.

### *C06—TRAUMATOLOGY 2*

### Treatment of proximal fractures of the humerus with Philos plate by deltopectoral approach

C. Iacobellis, C. Chemello*, R. Aldegheri

Clinica Ortopedica (Padua, IT)

**Introduction** Displaced proximal fractures of the humerus, with 3 or 4 fragment according to Neer, present several serious problems: complexity of proper reduction; precarious purchase of screws in the spongy bone of the head of the humerus, which has a very low bone stock; and risk of necrosis of the head, due to terminal vascularisation [1]. We analysed our results and failures for a critical assessment of the method used for repair.
**Materials and methods** We present results in 30 cases of proximal humeral fractures, 17 with 3 parts and 13 with 4 parts, treated with Locking Compression Plate (LCP, 14 cases) and Philos plate (16) by the deltopectoral approach[2]. Mean follow-up time, was 21 months. Patients were checked with standard X-rays and clinical evaluation, according to the Constant-Murley shoulder score, Individual Constant Score and Relative Constant score. The neck/shaft angle was measured according to Hertel et al.
**Results** All fractures were consolidated and no infections occurred. The mean Constant-Murley shoulder scores were Pain 10.6 (3–15), Activity of daily living 15.3 (2–20), Range of motion 26.8 (12–40) and Power 10.3 (3–25) and Total 63 (25–97). The Individual Constant score was 68.6% (27–98%) and the Relative Constant score 85.4% (36–130%). Fractures in 3 parts had a mean Constant score of 69.1 (17 cases), but this fell to 55 (13 cases) in those in 4 parts. Late necrosis of the humeral head occurred in two cases, both with 4-part breaks.

**Discussion** The deltopectoral approach offers good exposure and is especially recommended in 4-part fractures. The osteosyntesis must be stable if early mobilization of the shoulder and proper recovery of range of motion are to be achieved. As well as reduction and stabilization of the tubercles, it is also important to restore the neck/shaft angle and stabilize it with oblique screws fitting the plate to avoid varus malposition.

**Conclusions** The Philos plate resulted to be a good instrument to perform an excellent reduction and reconstruction in 3-part fractures. We thus believe that 3-part fractures, in which both reduction and stable osteosynthesis are easier, show favourable prognosis and should be clearly distinguished from 4-part ones.

**Suggested readings**

1. Klitscher D, Blum J, Andreas D, Hessmann M, Kuechle R, Du Prel JB, Rommens PM (2008) Osteosynthesis of proximal humeral fractures with the fixed angle Philos-plate. Eur J Trauma Emerg Surg 1:29–36
2. Babst R, Brunner F (2007) Plating in proximal humeral fractures. Eur J Trauma Emerg Surg 4:345–356

### Telegraph intramedullary blocking nail system in proximal and diaphyseal humeral fractures

N. Ruggieri*, G. Pristerà, C. Palumbo, G. Gargiulo

SCDO Ortopedia e Traumatologia, AOU San Giovanni Battista Molinette (Turin, IT)

**Introduction** The Telegraph intramedullary blocking system is a multi purpose system that allows humeral stabilization in proximal and diaphyseal secondary tumors and in fractures of the AO type 11 and 12. The short and long nails, through proximal humeral approach, are lowered 0.5 cm under the cortical line, proximally and distally anchorated by screws drived by a dima. This experience is gained in the last 5 years.

**Materials and methods** From January 2006 to December 2010, 204 Telegraph nails were used in 195 patients (9 bilateral cases), mean aged 57.1 years. In 38 secondary bone tumors, 19 short nails (10 fractures, 9 osteolisis) and 26 long nails (11 fractures, 15 osteolisis) were implanted. In 157 fracture 82 short nails (37 fractures 11A, 27 11B, 18 11C) and 77 long nails (27 12A, 32 12B, 18 12C) were implanted. Surgery was minimally invasive, except 25 cases in which a surgical opening on fracture level was required. The mean follow up of these patients was 17 months.

**Results** In tumors bone localization, the intramedullary nail allowed free mobilization in 42 on 45 humeri. In the humeral proximal fractures, valued with Constant score, a mean score of 77.8 points was reached with a 80.4% of good results. In the diaphiseal fractures, radiographic bone consolidation was reached in 7 weeks, full functional resumption in 8–9 week. We complained 4 consolidation delays (2 consolidation after 4 and 10 months, 2 undergone to new surgical treatment), 7 deficits in the elevation of the limb (4 cutting off of the nail treated by removal of the nail after bone consolidation), 6 fractures of the blocking screws that however allowed bone consolidation and 10 cases of necrosis of the humeral head treated by shoulder prosthesis.

**Discussion** The Telegraph nail system, with a minivasive surgery, allows an immediate stability and a suddenly recovery. Despite a proximal surgical approach, the nail caused no troubles to the rotatory cuff. Functional or bone consolidation troubles can be avoided by a careful technique. The humeral head necrosis, in the elderly patient, on one side doesn't create a significant reduction of the functional activity, from the other not even the shoulder prosthesis is able to reach a more complete function.

**Conclusions** The Telegraph nail system is reliable and allows an effective stabilization on traumatic pathologies and euplastic humeral diseases.

### Cement augumentation method for proximal epiphyseal humeral fractures in osteoporothic elderly patients treated by intramedullary nailing and plates and screws: a 1-year follow-up

C. Dall'Oca*, T. Maluta, P. Bartolozzi

Clinica Ortopedica e Traumatologica, Policlinico G.B. Rossi (Verona, IT)

**Introduction** Proximal epiphyseal humeral fractures are common in elderly people and their treatment has a rate of complications due to technical failure (cut out—head rotation).

**Materials and methods** We studied 12 patients (7 females, 5 males) with an average age of 76.37 years (range, 68–80 years) and severe osteoporotic bone (1 or 2 Singh score). They all had proximal epiphyseal humeral fracture, classified as type II-III following Neer classification, defined as fractures of the surgical humeral neck. Half patients were treated with T2 proximal humeral nail and half with Philos plate with a modified technique consisting in augmentation of the proximal screws. Augmentation was done with The Locker system (Tecres S.P.A.) inserted through the tunnel for the cephalic screws. The evaluation is based on: operating time, early functional recovery using the Costant–Murlay score, X-ray evaluation, mechanical and biological complications.

**Results** The Costant–Murlay average score was 70.1 after 1 month post-operation, 71.9 after 3 months, 73.2 after 6 months, 76.6 after 12 months. No other complications (infection, cut out and humeral head necrosis) were reported at the follow-up.

**Conclusions** Literature review and the observations based on the reported results allow to conclude that the cement augmentation of proximal screws in severe osteoporotic bone treated with T2 nail and Philos plate could improve the mechanical stability of the implant, ensuring early functional recovery.

### Treatment of nonunion of the humerus: our experience

G. Allevi*, M.A. Natale, T. Scuccimarra, S. D'Onofrio, V. Salini

Clinica Ortopedica e Traumatologica, Università "G. D'Annunzio" (Chieti, IT)

**Introduction** The post-traumatic pseudoarthrosis of the long bones continues to be one of the most complex diseases to treat. It is important to identify local and general, mechanical and biological risk factors which led to the failure of the healing process.

**Materials and methods** From January 2006 to January 2010, 10 post-traumatic diaphyseal aseptic nonunion of the humerus, classified with the NUSS (Non Union Score System) were treated at our Department. Five patients (males) with NUSS II (score between 26 and 50) without or minimal "bone defect" were treated with internal fixation and rh BMP7 (OP1.) The remaining five patients (3 males and 2 females), belonging to the III group NUSS (score between 51 and 75) and the presence of "bone defect" between 4 and 6 cm, were treated with plate, bone graft strut opposed and synthetic bone substitutes. The follow-up at 1, 3, 6, 9 and 12 months included radiographic and clinical evaluation (Constant Shoulder Score and VAS).

**Results** The five patients in Group II NUSS with a mean follow-up to 12 months showed a CSS between 11 and 17, mean VAS 3 and radiographic signs of complete healing at 6 months. The five patients of Group III NUSS with follow-up to 6 months showed a CSS between 9 and 16, mean VAS of 4 and radiographic signs of gradual recovery early as 3 months.

**Discussion** The pseudarthrosis is a condition where the bone healing is stopped. To restore this mechanism and 'a necessary stimulus osteoconductive, osteoinductive and osteogenic able to ensure mechanical stability and restart the physiological bone healing. The NON-UNION SCORE SYSTEM (NUSS) (2008) considers not only the bone stock and any problems related osteosynthesis, but also general clinical conditions of the patient and the status of soft tissue. THE "Diamond Concept", which was codified in 2007, also emphasizes the different mechanical and biological criteria, the latter distinguished by the cells, scaffold and growth factors.

**Conclusions** In our experience the use of bone morphogenetic proteins, synthetic bone substitutes and by homologous bank bone graft was indispensable to promote bone healing, providing a significant contribution even in the diaphyseal nonunion of the humerus, classified by NUSS, with critical bone defects.

### *C07—TRAUMATOLOGY 3*

### Functional outcome and short-term mortality after surgery for hip fractures

M. Mariconda*[1], M. Misasi[2], G.G. Costa[1], S. Cerbasi[1], G. Balato[1]

[1]Dipartimento di Chirurgia Ortopedica e Traumatologia, Università Federico II (Naples, IT);
[2]I Divisione di Ortopedia e Traumatologia, A.O.R.N. Cardarelli (Naples, IT)

**Introduction** Hip fractures are associated with significant disability and mortality. We conducted a prospective study to investigate the mortality rate and functional ability in patients who underwent surgery for hip fracture over a one-year period.

**Materials and methods** One-hundred patients (72 females and 28 males; mean age, 78.2 years (range 32–102) surgically treated for hip fracture were prospectively followed-up for 1 year. Fifty-five and 45 patients sustained a trochanteric fracture or a fracture of the femoral neck, respectively. Daily-life activities (ADL scale), cognitive impairment (MMSE Scale), comorbidity (CIRS scale), functional status, and personal mobility were prospectively evaluated 4 months and 1 year after the operation by telephonic interviews with the patient, his/her relatives, or primary care providers. Mortality data was also recorded. Possible outcome predictors were evaluated by regression analysis.

**Results** The overall mortality rate at 1 year was 19 percent. Male sex, comorbidity, and a poor pre-fracture functional status were significant predictors of mortality. ADL scale and personal mobility significantly improved between 4-month and 1-year follow-up controls. Forty-five percent of patients without preoperative walking limitations dropped out their walking aids 1-year postoperatively. Patients with trochanteric fracture treated with hip prosthesis showed greater and faster postoperative functional improvement with respect to patients treated by gamma nail.

**Discussion** Current results represent the first report of a wider ongoing prospective study aimed to evaluate possible outcome predictors of hip fractures to be used to improve and personalize the surgical treatment. The preoperative functional status and comorbidities are major determinants of 4-month and 1-year postoperative mortality and ability of subjects.

**Conclusions** Our preliminary findings demonstrate the role of surgery in promoting the functional improvement of patients who sustained hip fracture.

### Hybrid external fixation in distal femur fractures: our experience

G. Corina*[1], A. Marsilio[1], N. Tartaglia[2]

[1]U.O.C. Ortopedia, P.O. "Vito Fazzi" (Lecce, IT);
[2]U.O.C. Ortopedia, P.O. "San Paolo" (Bari, IT)

**Introduction** Distal femoral fractures represent the 8% of knee fractures mostly occurred in high energy trauma in young patients. Internal fixation and/or endomedullary fixation are the treatment of choice. We present our experience treating distal femoral fractures with hybrid external fixator.

**Materials and methods** From January 2005 to December 2010, 14 patients (11 males and 3 female; 9 were children and 5 were associated to a leg fractures). Fractures were classified according to OA classification, Gustilo–Anderson classification, Tcherne classification. The minimum follow-up was 24 months and results were assessed according to Neer-Grantham-Shelton table.

**Results** All patients healed and no cases of infections, non-union or necrosis were observed.

**Discussion** The advantages of this technique are: a quick operation made as close surgery (with minimal internal fixation associated where indicated and possible), minimal blood loss, chance to extend the external fixation to the leg with few morses, minimal change of mobility.

**Conclusions** According to literature, authors believe that hybrid external fixation in distal femoral fractures is a treatment of choice in open fractures, in fractures associated to soft tissue injuries and in children fractures. It is a surgeon's choice in the remaining cases.

### Intramedullary nail for the treatment of pseudoarthrosis of the tibia and femur

M. Di Liddo*[1], D.M. Donati[1], L. Cevolani[1], T. Frisoni[1], S. Giannini[1]

Rizzoli Orthopaedic Institute (Bologna, IT)

**Introduction** Pseudoarthrosis is a fracture that after 9 months from the trauma cannot heal spontaneously, it is not consolidate and does not have clinical and radiographic signs of consolidation. The psuedoarthrosis represents the failure of the reduction systems and of the osteosynthesis. It leads to the lack of progress of the callus in bone repair and interposition of fibrous tissue between the stumps.

**Materials and methods** During the last 10 years, at the Rizzoli Orthopaedic Institute in Bologna, we treated 25 patients with intramedullary nail (17 have an aseptic pseudoarthrosis of the tibia and 7 of the femur). In the femure, all the fracture that lead to pseudoarthrosis were not exposed. The ostesynthesis of pseudoarthoris was performed with locked nail in 6 cases and with dynamic nail in 2 cases. In the tibia, 7 cases presented exposed fracture at the beginning, the remaining 11 cases the fractures were not exposed. At the time of treatment in all cases there were no clinical and laboratory signs of active infection. Ostesynthesis was performed with intramedullary nail in 14 cases and with locked nail in 3 cases.

**Results** In the femoral pseudoarthrosis, in 2 cases ostesynthesis did not lead to healing. In 6 cases the intramedullary nail led to healing after an average of 11 moths. In the pseudoarthrosis of the tibia, in 2 of the cases who presented exposed fracture at the beginning we saw a resumption of infection and in 1 case the removal of the intramedullary nail and the application of external fixation was necessary. The consolidation in nonunions of the tibia treated with intramedullary nail occurred in 16 cases and the average time of healing was 5 months.
**Conclusions** The intramedullary nail is one of the most used fixations for the treatment of pseudoarthrosis of the femur and of the tibia as far as the simplicity of the surgery technique is concerned and the versatility as well. In our series we found good results in the pseudoarthrosis of the tibia where the consolidation occurred in 16 of 17 cases treated by intramedullary nail. In all the cases, the functional recovery and the load were early and the tolerability of the intervention and the fixation were good. In the femur, the results were not so good because of the higher complexity of nonunion treated and of the too rigid fixation.

### Distal femur and distal tibia fractures treated with MIPO technique: mid-term outcomes

L. Di Giorgio*[1], P. Braidotti[2], G. Touloupakis[3], E. Theodorakis[3], L. Sodano[3], G. Cassisa[2], G. Baumgartner[2], C. Villani[1]

[1]Ortopedia e Traumatologia B, Dipartimento Scienze dell'Apparato Locomotore, Università di Roma "Sapienza" (Rome, IT);
[2]UOD Traumatologia Ortopedica, DEA II Livello, Policlinico Umberto I (Rome, IT);
[3]Scuola di Specializzazione in Ortopedia e Traumatologia, Università di Roma "Sapienza" (Rome, IT)

**Introduction** MIPO is a minimally invasive osteosynthesis technique providing a relative stability biological plating in which blood supply to the fractured fragments and soft tissues is maximally preserved. The objective is to provide an anatomic reconstruction of the articular surface, a correct femoral/tibial axis alignment and a stable fixation, leading to an early functional/range of motion restore.
**Materials and methods** Between February 2009 and December 2010, at the University Hospital of Rome "Sapienza" we treated 25 patients (28 fractures) with distal femur and distal tibia fractures using the MIPO technique. The mean age of the patients was 50 years (13 female, 12 male), 21 with distal femur and 7 with distal tibia fractures. AO classification system was used. Minimum follow-up was 6 months (6–20). Clinical assessment was performed at 3, 4, 6, 12 and 20 months after surgery evaluating range of motion and according to Lysholm and Olerud/Molander questionaire. Radiographic assessment was performed post-operatively and consequently every 2 months up to 1 year using the standard AP and LL views. Damage control and multiple stage surgery was used when necessary.
**Results** The mean healing time was 12 weeks. No significant varus/valgus deformities or rotational defects were detected. We observed very good clinical outcomes with high range of motion and complete weight bearing after bone healing. A high Lysholm and Olerud/Molander questionnaire score was obtained. There were no cases of pseudoartrosis, superficial/deep infection, implant failure or hardware impingement. To date none of these implants was removed.
**Discussion** Satisfactory results with ORIF were reported in the past, using conventional plating, but rate of reintervention for complications such as pseudoartosis, loss of reduction and implant failure was high. Several studies report very good/excellent results using the MIPO "bridging" tecnique, with a low rate of complications.
**Conclusions** Our experience confirms the validity of the technique.

### Dilops: the new divergent locked screws system for treatment of pertrochanteric fractures

P. Palombi*, A. Palombi

(Rome, IT)

**Introduction** The divergent locked screws system for the treatment of pertrochanteric fractures (AO/OTA 3.1.A1 and A2) is introduced: it is characterized by the biomechanical advantage of the shortening of the lever arm that stands between the gravitary axis bearing on the femoral head and the fulcrum of the osteosynthesis system, allowing a robustness osteosynthesis reached by mini-invasive approach. Surgical tecnique is shown. Aim of this paper is to evaluate the new system and compare its preliminary results with the current literature. At the time of this paper it is possible to comment only preliminary results.
**Materials and methods** From July 2009 to January 2011, we treated 140 proximal femoral fractures (AO/OTA 31.A1 and 31.A2) with divergent locked screws system (96 women and 44 men). One hundred and twenty of them (76 women and 45 men) were included in the follow-up term of 1, 3 and 6 months.
**Results** All patients had good or excellent midterm results evaluated by objective and subjective scores. Objective scores were assessed by orthopaedic surgeons on the basis of clinical and radiographical assessment, intra- and post-operative blood loss, operative time, intraoperative X-ray exposition, rate of complications and failure, time to the full weight bearing. Subjective scores were assessed by surgeons and patients on the basis of restored function, restored quality of live and overall satisfaction.
**Discussion** The divergent locked screws system showed to be a powerful and cost-effective alternative in the treatment of stable (AO/OTA 31.A1) and unstable (AO/OTA 31.A2) pertrochanteric femoral fractures. It showed easy intraoperative management of fracture fragments, very good stability, and rapid bone healing.
**Conclusions** The strength of this system seems to be the favorable biomechanics of the load bearing on the lower arm, achieved by a reasonable use of locking plates. Despite the use of very small and light materials, it obtain the most suitable advantages of both intramedullary and extramedullary systems, like easy intraoperative control of the fragments, respect of soft tissues and reduction of blood loss, leading to an elastic osteosynthesis, and shortening of the operative and healing times. We are keeping register ring new results for a more extensive evaluation and accurated study.
*Type of study*: prospective, multicentric, non-randomized.

### *C08—TRAUMATOLOGY 4*

### Analysis of time hospitalization for treating hip fractures and correlation with the American Society of Anesthesiologists Classification of Medical Comorbidities: our experience

F. Valli*, R. Giancola, C. Crippa, G. Antonini

AO "San Carlo Borromeo" (Milan, IT)

**Introduction** Fracture of the hip is the second leading cause of hospitalization in the geriatric population and the ward cost contributed 84% of the total hospital expenditure per patient. Some factors, such as preoperative comorbidities and postoperative complications, have a role in time of hospitalization. The American Society of Anesthesiologists

(ASA) classification system may be a useful risk-stratification system for elderly patients who sustain a hip fracture. The aim of this study was to analyse the time of hospitalization. We hypothesized that the ASA class would be a predictor of common medical problems occurring in the immediate postoperative period after a hip fracture surgery and this is useful to predict the time of hospitalization.
**Materials and methods** We analysed 43 patients, 15 men and 28 women, the average patient age was 81.9 years (96–66), treated with endoprosthesis from January to June 2010. A retrospective chart review was performed to obtain data regarding length of hospital stay, medical and surgery complications, time to surgery, time between request of exams and their execution and request of transfer in rehabilitation ward and discharge. All patients were assigned an ASA class as part of their anesthesia preoperative assessment.
**Results** The average days of hospitalization was 16.9 (38-8), 14.6 in ASA class-2, 16.7 ASA-3, 32 ASA-4; the average days to surgery was 7.4 (21-1), 5.1 ASA-2, 7.5 ASA-3 and 20 ASA-4; the average post-operative days was 7.5 (18-2), 7.5 ASA-2, 7.1 ASA-3 and 10 ASA-4; the request of exams was 33.3% in ASA class-2, 68.1 % ASA-3 and 100 % ASA-4; the average days between request of transfer and discharge was 5.5 (17-1).
**Discussion** Our data suggest to stratify patient risk on the basis of the ASA class in the emergency department and this is the key to hospitalize, in fact if the patient is in ASA class-2, postoperative complications are more likely to be surgical or rehabilitation-related, if the patient in ASA class-3 or 4, there is a higher risk that the patient will have a medical complication postoperatively. Consequently it is more correct an admission to a medical service.
**Conclusions** In conclusion, ASA class correlates with perioperative medical problems following hip fracture surgery, and patients who sustain a hip fracture require a multidisciplinary vision to care and an “orthogeriatric” approach is optimal choice.

### Proximal femur fractures in the elderly: social impact and mortality index a an year with surgical treatment

F. Nicolosi*, W. Leonardi

U.O. Ortopedia e Traumatologia, AORNAS Garibaldi, Presidio Garibaldi Nesima (Catania, IT)

**Introduction** The femur proximal fractures in the elderly represent a social health problem remarkable in the industrials countries like Italy. In this paper we display the mortality index and main associate factors.
**Materials and methods** We evaluated all patients with an age >65 years operated in 2010. We compared the raw index of mortality in both genders in the 12 months following surgical treatment; the mortality rate increases with the increasing age together with anaesthesia risks and associated diseases.
**Results** The mortality index is also hindered by the surgicals instruments of synthesis; the localization of the fracture (medial or lateral); the reduction of the cognitive functions; the interval between trauma and surgical treatment larger than 48 h.
**Discussion** The achieved results may presume the possibility of selection of some categories with majory risk of mortality in the first year after surgical treatment. For these patients we must evaluate the need of particulary care in the surgical treatment and in the post-surgical rehabilitation.
**Conclusions** This work show the relevant social health impact in modern society of proximal femoral fractures especially considering the age increase in general population, thus a particular attention has to be given to the treatment of this disease problem.

### Comparing osteosynthesis techniques in periprosthetic femoral fractures: considerations on personal cases

M.A. Rosa*[1], G. Topa[2], G. Caruso[1], P. Ditto[1], S. Barreca[1]

[1]Sezione Ortopedia e Traumatologia, Dipartimento Specialità Chirurgiche, Scuola di Specializzazione in Ortopedia e Traumatologia, Università degli Studi di Messina (Messina, IT);
[2]UOC Ortopedia e Traumatologia, Azienda Ospedaliera “Bianchi-Melacrino-Morelli” (Reggio Calabria, IT)

**Introduction** The hip prostheses are often implanted in patients with poor bone quality. Even if the implant may solve the fracture or degenerative problems, nothing can be done against any injury that may occur in the periprosthetic region, where the presence of a metallic structure may be valid to create a “locus minoris resistentiae” in the surrounding areas, resulting in a failure of a largely porotic bone structure.
**Materials and methods** The reported experience examines 12 patients with a periprosthetic fracture of the femur treated with different techniques. Plate, screws and cerclage in five cases, retrograde nail in four cases, external fixator in one case and in two cases long stem implants for revision surgery were used. The age is between 67 and 89 years old and referring to sex six were males and five females.
**Results** The surgical results were assessed by postoperative radiographs and functional results were evaluated by considering the functional recovery related to age and the way of living and the expectations of the patients before the fracture. The ‘Harris Hip Score’ and the ‘Knee Score Index’ were applied in all cases.
**Discussion** The choice of osteosynthesis tecniques was motivated by several factors: patient age, general conditions, type of periprosthetic femoral fracture, functional needs of patients, surgeon’s knowledge of different techniques. A Diaphyseal fracture, few centemeters away from the femoral stem prosthesis, in a patient with aseptic loosening, allowed a system of review with a longer stem because of the good general condition of the patient and his request for a good functional level. A relatively young patient (67 years) was treated in two stages: the acute stabilization of fracture with plate, screws and cerclage was followed, 8 months later, by an operation with a prostethic revision implant.
**Conclusions** The good results obtained “by customizing the treatment” based on the type of fracture, patient’s conditions and his functional requirements point out the importance of the surgical planning in dealing with this particular type of fractures.

### Fractures of the distal femur in elderly patients: osteosynthesis or prosthesis?

P. De Biase*[1], D.A. Campanacci[1], E. Biancalani[1], P. Caldora[2], A. Piccioli[3], R. Capanna[1]

[1]AOUC (Florence, IT);
[2]Ospedale San Donato (Arezzo, IT);
[3]CTO (Rome, IT)

**Introduction** The increase in life expectancy and the level of activity performed by elderly patients has produced an increase in high-energy fractures in these patients. Particularly in the distal femur, articular and metaphyseal fractures in these patients pose complex choices about the type of treatment. Our goal is to evaluate the results obtainable with osteosynthesis or with a prosthesis in a very selected group of patients.

**Materials and methods** We selected patients at an age of greater than 65 years treated at our Department with distal articular or periprosthetic fractures of the distal femur. These patients were evaluated regarding the type of treatment, operative time, previous functional status, functional status at the last follow-up. We identified 10 patients with fractures of the distal femoral joint, or on pre-existing knee prostheses treated with an internal fixation. In 9 cases a titanium plate with angular stability was used, and in one case a retrograde femoral nail was used. We identified 10 patients whose articular distal femur fracture was immediately treated with a prosthetic modular knee. In all cases megaprothesis of Waldemar Link (Megasystem C) were implanted.
**Results** At an average follow-up of at least one year we reviewed the patients. Patients treated with osteosynthesis had returned on average to a full weight-bearing 4 months after surgery, compared with 40 days for patients treated with a prosthesis, ROM was 87° in patients treated with osteosynthesis with an average extension deficit of 5°, while in patients treated with prosthesis was 95°, with an average deficit of 2° extension. Symptomatic limitations on walking more than 500 meters and the need for support was present in 6 out of 10 patients with synthesis. In patients treated with implants only 3 required the support of a walking aid. We have no recorded cases of infection in this series of patients.
**Discussion** Treatment of fractures of the distal femur in the elderly with prosthesis can be considered in our opinion a good way to rehabilitate these patients providing a quick, stable and permanent solution to their trauma. The subjective and objective results in this select group of elderly patients were better for prostesis treated patients. The long-term results of the megaprosteses are not available in this series, but the long experience in patients with cancer, much younger and more active, gives us confidence in this regard.

### Intramedullary nailing plus wire cerclage in femoral fracture: our experience

S. Carta*, M. Fortina, D. Scipio, A. Riva, S. Di Giacinto, P. Ferrata

Ortopedia Universitaria, AOU Senese (Siena, IT)

**Introduction** The cortical wire cerclage have been saw like the devil by the ortopedics however which have been an easy practice in bone cylinder discontinuity reduction. Our experience in hip arthroplasty femoral revision surgery convinced us to introduce the wire cerclage in femoral fractures associating it with intramedullary nailing alternatively to traditionally more invasive and less biomechanically advantageous plate and screw osteosinthesis.
**Materials and methods** We have started to use this technique in femoral fractures in 2008. We had treated 13 patients in whom the wire cerclage was needed in fractures reduction with intramedullary nailing.
**Results** We had very good results in fractures healing and patient function recovery without complications.
**Discussion** The use of the cerclage wires associated with intramedullary nailing has long been deprecated although largely by word of mouth, as there is little in the literature to support or disclaim this stance. Certainly Charnley (1961) decries the evil effects of circumferential suture of bone on the grounds that it could devitalize bone fragments and prevent the extension of periosteal callus. More recent studies of the blood supply of mammalian long bones have shown that the periosteal blood vessels do not run longitudinally but enter the cortex tangentially (Rhinelander, 1968) and a circumferential cerclage wire with its small-area cortical contact is the least likely to interfere with this cortical blood supply. More recent experiences in prosthesis stem revision procedure allowed to confirm the good results in the association between intramedullary device and cerclage wiring. Infact our experience in this practice convinced us to extend the use of cerclage wires in intramedullary nailing allowing: optimal multi-fragment fractures reduction; improving fractures stability also expanding the indications for intramedullary nailing.
**Conclusions** The intramedullary nailing sinthesys is less invasive, high biomechanical stable but presents more difficulties in fractures reduction mainly with 3 or more fragments. Using cerclage wires is possible to bridge this problem with advantages in terms of low invasivity, bimechanical stability and rapid recovery respect to plate traditional synthesis.

### Cement augmentation method for intertrochanteric fractures in osteoporothic elderly patients treated by intramedullary nailing: a 3-year follow-up

C. Dall'Oca*, T. Maluta, P. Bartolozzi

Clinica Ortopedica e Traumatologica, Policlinico G.B. Rossi (Verona, IT)

**Introduction** Trochanteric fractures are common in elderly people and their treatment has a rate of complications due to technical failure (cut out-head rotation);
**Materials and methods** We studied 27 patients (15 female, 12 male) with an average age of 85.16 years (range, 80–94 years). They had an unstable trochanteric fracture, defined as fractures with three fragments or more, age more than 80 years and severe osteporotic bone (1 or 2 Singh score). All patients were treated by Gamma Nail standard technique and augmentation was done with The Locker system (Tecres S.P.A.) inserted through the cannulated cephalic screw at its apex. The evaluation is based on: operating time, early functional recovery using the modified Harris hip score, Rx TAD and sliding screw, mechanical and biological complications. During the follow-up we lost 7 patients. No one died because of surgical and anesthesiological complications or other causes due to the surgical procedure.
**Results** The HHS average score was 49.4 after 1 month post-operation, 57.31 after 3 months, 55.8 after 6 months, 59.25 after 12 months, 61.67 after 24 months and 61.55 after 36 months. The average decrement of haemoglobin was 1.58. In one case we reported a cement migration through the femoral head. No other complications (infection, cut out and femoral head necrosis) have been reported at the follow-up.
**Conclusions** Literature review and the observations based on the reported results allow to conclude that the cement augmentation in severe osteoporotic bone could improve the mechanical stability of the implant, ensuring early functional recovery.

### *C09—SHOULDER AND ELBOW 1*

### Anterior dislocation of shoulder prosthesis: a clinical case

M. Palumbo*, P. Paladini, F. Campi, G. Porcellini

U.O. Chirurgia Ortopedica della Spalla (Cattolica, IT)

**Introduction** Anterior dislocation of a Total Shoulder Arthroplasty (TSA) and of Hemi Shoulder Arthroplasty (HSA) is a rare but important complication. The incidence of this complication is from 0 to 29% with a 2.8% mean. Superior dislocation is usually due to a cuff insufficiency, the inferior dislocation to a too low position of the humeral component. Anterior and posterior dislocation are frequently due to a subscapularis failure or to an excessive anterior or posterior version of the humeral head.

**Materials and methods** We describe an atypical case of a chronic dislocation of a HSA in a paraplegic patient with massive cuff tear. The patient experienced an anterior glenoid rim fracture that made impossible the implant of glenoid component leading to a HSA implant. Following surgery the HSA dislocated anteriorly lying on brachial plexus.
**Results** We treated the patient after 6 months from previous surgery. The HSA was dislocated under the conjoint tendon with complete detachment of the subscapularis from glenoid neck and scapula. To remedy to the anterior glenoid bone loss, about 30%, we transposed coracoid process as in a Latarjet procedure obtaining a good surface to implant an un-cemented glenoid component of a Reverse Shoulder Arthroplasty (RSA). At 1-year follow-up the patient recovered a full range of motion, as compatible with RSA.
**Discussion** Anterior dislocation of a shoulder prosthesis is a challenge for shoulder surgery for the poor quality of soft tissues and osteoporotic bone. The use of an iliac crest graft of coracoid process is mandatory in these cases.
**Conclusions** In case of revision of a dislocated shoulder prosthesis, it is important to set up the patient in operating theatre to make possible the use of an iliac crest or coracoid process graft in case of anterior glenoid bone loss.

### Tuberosities healing in anatomical hemiprosthesis for shoulder fracture

A. Tucci*[1], D. Vanni[2], A. Silvestro[1], S. Lorini[1], V. Salini[2], R. Angeloni[3], R. Tucci[3]

[1]Villa dei Fiori (Naples, IT);
[2]Università D'Annunzio (Chieti, IT);
[3]AOU Careggi (Florence, IT)

**Introduction** The clinical outcome of shoulder hemiprosthesis for fracture strictly correlates with tuberosities healing [1]. Different prostethic models were designed to promote tuberosities healing without success [2]. Aim of the study is to report the 1-year clinical and radiological findings in patients treated with an anatomic shoulder hemiprosthesis (Zimmer®, Warsaw, USA) specifically designed for fracture, characterized by a large metaphysis covered by spikes, which should improve the stability of tuberosities reconstruction.
**Materials and methods** 35 patients operated in three different Italian Hospitals with a Zimmer shoulder fracture hemiprosthesis were included in this study. Patients were prospective followed-up with a Constant score and a plain X-rays examination at 6 and 12 months. Age, sex, fracture type and the time interval between fracture and operation were recorded. Tuberosities resorption, malreduction and/or dislocation, as well as humeral lateral offset, acromion-humeral distance and the distance between the highest part of the humeral head and upper part of greater tuberosity (head height) were analyzed.
**Results** One patient developed a periprosthetic fracture 2 months after surgery and was excluded from the study, thus, a total number of 34 patients were followed-up. A complete 12 months clinical and radiological follow-up will be completed in September 2011 and will be presented at the S.I.O.T. Congress for the first time. The average Constant score at 6 months was 68 points. The 6 months mean elevation value was 130°. Partial/complete tuberosities resorption was observed in 9/2 patients (26%/6%) at 6 months.

**References**
1. Robinson CM, Page RS, Hill RM, Sanders DL, Court-Brown CM, Wakefield AE (2003) Primary hemiarthroplasty for treatment of proximal humeral fractures. J Bone Joint Surg Am. 85:1215–1223
2. Loew M, Heitkemper S, Parsch D, Schneider S, Rickert M (2006) Influence of the design of the prosthesis on the outcome after hemiarthroplasty of the shoulder in displaced fractures of the head of the humerus. J Bone Joint Surg Br 88:345–3450

### Surgical repair of the rotator cuff

F. Specchiulli, M. Rendina*, M. Berardi

Cattedra di Malattie dell'Apparato Locomotore, Università degli Studi di Foggia (Foggia, IT)

**Introduction** The repair of the rotator cuff is one of the most common surgical techniques in use at least since two decades. However, which method is the most appropriate is still debated because the orthopedic surgeon can choose between three tecnique: open, arthroscopic and mini-open.
**Materials and methods** The study includes 34 patients, mean age 55 years, undergoing open surgical repair of the rotator cuff. The cuff lesions were classified according to Patte in distal, intermediate and proximal. In 90% of the cases were isolated lesions of the supraspinatus or infraspinatus associated with, in 10% isolated lesion of the subscapularis. In 7 patients, the CLB had signs of tendinosis more or less advanced in 3 was absent. MRI gave particular importance to the presence of fatty infiltration of the muscles of the cuff following the classification of Goutallier et al.
**Results** Results evaluation was performed by using the Constant-Murley score, the VAS score and the UCLA score. At an average follow-up of 28 months (12 m/36 m), all patients underwent ultrasound examination of the shoulder in order to assess the integrity of the surgical repair at a distance. The average Constant-Murley score increased from 52.8 p. preoperatively to 79 p. at control time. The average UCLA score was 31 p. with an improvement in terms of pain, function and movement. The average VAS score at rest increased from 7.5 (average pain—severe) to 1.7 (no pain). Ultrasound monitoring showed recurrence of the lesion in 10 patients.
**Discussion** Recent studies have shown that the results of open repair are completely overlapping, the same apllies to the results of the arthroscopic technique to repair mini-open. The clinical and radiographic data show that the fixation with double-row suture anchor allows a more solid anchoring but also a more normal tendon healing. The recurrence rate appears to be related to poor quality of tendon tissue, the method of fixation, the size of the lesions, the fatty infiltration of muscles, inadequate postoperative protection.
**Conclusions** Since we do not know fully the pathogenesis and natural history of disease, remains controversial work when and what type of intervention performed. This explains the need for further and more detailed studies to clarify the still unknown aspects of this disease. Future research will answer all the today unanswered questions.

### *C10—SHOULDER AND ELBOW 2*

### Treatment of proximal humerus fractures with locking plate by minimally invasive technique

F. Battistella*, M. Oldani, M.S. Tajana, D. Prestamburgo

Azienda Ospedaliera, Ospedale Civile Legnano (Legnano, IT)

**Introduction** The second generation of locked plating for treatment of proximal humeral fractures allows both open with deltopectoral approach and minimally invasive (MI) with percutaneus reduction and anterolateral deltoid split approach. Recent anatomical studies evidenced potential advantages of MI technique, with better preservation of soft-tissue and a lower risk of AVN. Aim of our study was to evaluate the clinical and radiological results of the two different surgical approaches. Study design: prospective and randomized clinical study.

**Materials and methods** Between January 2007 and March 2010, 54 consecutive patients (38 female, 16 male, mean age 61 years) were included in this study. Inclusion criteria were displaced two-part surgical neck fractures, or three-part valgus impacted fractures. Exclusion criteria for the study were pathological fractures, open fractures, and age less than 18 years and more 75 years. The patients treated with ORIF by standard deltopectoral approach were 26, and treated with MI techinique were 28. The implant used was a second generation of locked titanium plate (NCB-Zimmer, Inc.) anatomically precontoured with four or five hole on the plate shaft. Screws can be inserted with a radius of 30° (polyaxial screw placement). The MI technique is based on anterolateral deltoid split approach and percutaneus reduction. A radiolucent aiming system is available for MI plate insertion, the plate is attached to the handle bar and inserted submusculary, mantening the distal plate tip on the bone to be sure to protect axillary nerve. The screws are inserted with a sleve protection assembly and a drill guide. The planned followup were at 1, 3, 12 months. At each follow-up, range of motion (ROM), VAS was determined and iADL score, Constant score was applied, standard X-rays were obtained. The results were subjected to statistical analysis. Results with $p < 0.05$ were considered statistically significant.

**Results** Statistically significant differences between two groups were seen for VAS, ROM, iADL and Constant score. X-rays showed healing of the fractures after 12 months in 49 cases (91%). Complications: intra-articular screw perforation in 6 cases (12%: 4 open 2 MI), secondary displacement in 4 cases (8%: 2 open 2 MI). In the MI group no clinical sign of axillary nerve lesions was detected.

**Discussion** The NCB-PH plate implanted with MI approach joins the biomechanical advantages of a second plate generation with a less traumatic and more anatomical surgical approach, with better clinical results.

**Conclusions** This study shows that MI approach is a valid option for the treatment of displaced proximal humerus fractures.

### Modified Du Toit-Roux open capsulorraphy for recurrent anterior shoulder instability: clinical, functional and radiological outcomes

A. De Carli, A. Vadalà*, C. Iorio, R.M. Lanzetti, G. Esposito, A. Ferretti

UOC di Ortopedia e Traumatologia, Ospedale S. Andrea, II Facoltà di Medicina e Chirurgia, Università di Roma “Sapienza” (Rome, IT)

**Introduction** Recurrent traumatic shoulder instability is a common finding among young active people and several surgical techniques were described. The aim of this study is to retrospectively evaluate clinical, functional and radiological results of young active patients treated with the modified Du Toit-Roux technique.

**Materials and methods** Fifty sport active patients with recurrent traumatic anterior shoulder dislocations, reporting a minimum of 3 episodes, surgically treated between January 2005 and September 2007 with the open capsulorraphy described by Du Toit and Roux and modified in our Department with the use of suture anchors, were included in the study. All patients underwent an accurate clinical exam (in order to determinate range of motion and muscle strength, using dynamometric test), evaluation scales (Rowe, Walch-Duplay, VAS) and a radiographic exam (both in antero-posterior and axial view).

**Results** At a medium follow-up of 48 months (range 40–60), we found one case of re-dislocation (2%); the medium loss of external rotation compared to the contralateral side was 7.8°; 46 patients (92%) had a negative apprehension test; mean results of the evaluation scales were excellent (Rowe 96.7 ± 11.6, Walch-Duplay 93.7 ± 11.8, VAS 0.2 ± 0.7); radiologically, we found 6 patients (12.5%) with mild glenohumeral arthritis (grade 1 according to Samilson-Prieto scale). No strength loss was found in patients treated on dominant side shoulder (35 patients) compared to the unaffected one, while in patients operated on non-dominant side (15 patients) a mild deficit (96.7% of the opposite side) was detected ($p > 0.05$).

**Discussion** Our study shows how modified Du Toit-Roux open capsulorraphy is a reliable technique for the treatment of recurrent anterior glenohumeral instability, with very low percentage of recurrence (2%), mild loss of external rotation (7.8°), mild or no strength loss in internal rotation at mid-term follow-up. Furthermore, no signs of moderate/severe arthritis were detected.

**Conclusions** Modified Du Toit-Roux open capsulorraphy yields to excellent clinical and radiographic results at mid-term follow-up.

### Subacromial viscosupplementation with sodium hyaluronate (SportVis) in rotator cuff tendinopathy: prospective controlled randomized study with rehabilitation therapy

G. Merolla*, G. Porcellini

U.O. Chirurgia Spalla e Gomito, Azienda AUSL di Rimini (Cattolica, Rimini, IT)

**Introduction** Rotator cuff tendinopathy is a common cause of pain and shoulder dysfunction. In the current study we compare the results of sodium hyaluronate subacromial injections with rehabilitation therapy.

**Materials and methods** We enrolled 40 patients (M/F: 24/16; mean age: 53 years; shoulder right/left: 31/19) with persistent shoulder pain for at least 4 months. Exclusion criteria were: rotator cuff tear, calcifying tendinitis, gleno-humeral instability, osteoarthritis, rheumatic diseases, physical therapy and/or injection in the previous 4 months, shoulder surgery, anesthetic nerve block, trauma, severe medical diseases. The included subjects were randomly enrolled in the *group A* (20 cases) who received 2 ecoguided subacromial injections (baseline and 14 days) and in the *group B* (20 cases) who underwent to rahabilitation therapy including active shoulder mobilization, soft tissue stretching and humeral positioners strengthening for 30 days (3 sessions every week). Clinical assessment of shoulder function was performed with VAS score for pain (0–100), Oxford shoulder score (OSS) and Constant-Murley score (CS). All patients were examined at baseline, 14 days, 30 days and 6 months. Statistical significance was set at 5% ($p < 0.05$).

**Results** Pain improvement in the *group A* was statistical significant at 14 days ($p = 0.0093$) and at 30 days ($p < 0.0096$). The mean score for pain improved at 6 months ($p = 0.0476$) but we registered worsening in pain score in 5 cases (mean VAS score: 65).

A significant difference was found with OSS ($p = 0.0389$) and CS ($p = 0.0401$) at all 3 follow-up. In the *group B* we registered significant difference at 14 days ($p = 0.0398$) for VAS score, OSS and CS while the results deteriorated at 30 days ($p = 0.0531$) and at 6 months ($p = 0.0547$) for all three clinical tools.
**Discussion** Subacromial injections with SportVis is more effective than rehabilitation therapy to treat pain and dysfunction of the shoulder due to rotator cuff tendinopathy.
**Conclusions** Good preliminary results of subacromial shoulder injection with SportVis encourage to extend their use in patients with symptomatic rotator cuff tendinopathy.

### "Univers" shoulder prosthesis in proximal humerus fractures

G. Logroscino*, G. Milano, V. Ciriello, C. De Ieso, V. Izzo, C. Fabbriciani

Università Cattolica del Sacro Cuore (Rome, IT)

**Introduction** The treatment of proximal humeral fractures is widely debated and depends on age, comorbidities, and bone quality. Prosthetic replacement is indicated for fractures with a high risk of necrosis. New specifically designed implants based on the refinement of surgical techniques and technologies should permit significant improvement in clinical outcomes than in the past. The aim of this study was to evaluate the effectiveness of the prosthetic treatment of shoulder fractures with a new type of trauma implant.
**Materials and methods** Twenty-five proximal humeral fractures were treated between 2005 and 2008, with hemiarthroplasty replacement. The fractures were classified based on X-ray (Neer's Trauma Series), 3D—CT according to the Neer's classification and Hertel's criteria. The patients were evaluated clinically (Constant Score, VAS) and radiographically at 1–3–6–12 months and then at annually follow-up (F-Up). A modular uncemented prosthesis (Univers-Trauma, Arthrex, Naples, FL) was implanted in all the cases. The average F-Up was 36 months (min. 24 months and max. 60 months).
**Results** The results were excellent\good in 8 cases, fair in 11 cases and unsatisfactory in 4 cases. A gradual improvement was observed up to a year (Constant = 79, VAS = 16 mm.). At the end of F-Up (36 months) the outcome was stable (Constant = 78.8; VAS = 15 mm.). Five complications were observed: three periprosthetic fractures and two infections (excluded from the study). The greater tuberosity was well positioned and consolidated in 65% of cases and reabsorbed or malpositioned in 35% of cases.
**Discussion** In the past Neer reported excellent results for arthroplasty substitution in shoulder fractures, but these results were never been confirmed afterwards. Currently, the functional recovery is poorly predictable and reproducible, ranging from 56 to 91%. Our series confirms these data, having observed good or moderate results in 82% of patients with pain relief in almost 92% of cases. Resorption (24%) and malposition (8%) of the tuberosities well correlates with 17% of unsatisfactory results.
**Conclusions** The treatment of proximal humeral fractures with hemiarthroplasty has shown a prompt improvement of joint function in the absence of pain. The results depend on the proper reconstruction of the joint geometry, with particular attention to the tuberosities. The new generations of prosthesis facilitate a better anatomical reconstruction, but the final result is almost always lower than normal and the indications are to be reserved to the strictly necessary cases.

### The traumatic posterior dislocation of the scapulo-humeral joint: evaluation of 35 cases

C. Rovesta*[1], M.C. Marongiu[1], M. Del Giudice[1], V. Duca[1], L. Celli[2], F. Catani[1]

[1]Clinica Ortopedica e Traumatologica, Dipartimento ad Attività Integrata di Patologie dell'Apparato Locomotore (Modena, IT); [2]Hesperia Hospital (Modena, IT)

**Introduction** The posterior dislocation of the shoulder is rare and caused by a violent trauma to the shoulder in adduction and internal rotation. Clinical and X-rays diagnosis is not easy and about 50% of cases is not recognized or recognized too late with a picture of inveterate dislocation, which makes it difficult to treat.
**Materials and methods** We controlled clinically (Constant and SST) and with rx, 35 patients (29 M and 6 F) mean age 48 years (min. 21–max. 86) treated at the Orthopedic Clinic of Modena for traumatic posterior dislocation of the shoulder from January 1996 to October 2010. A first group (A) of 19 patients, one bilateral, were observed and treated in emergency or within 3 weeks after the injury. 11 patients showed no associated fractures, and for them we performed reduction and immobilization in neutral rotation, 8 patients showed a fracture of the humeral head and after CT evaluation. We performed a synthesis with plate (4 patients), an endo prosthesis in 3 and an inverse prosthesis in one. The group (B) includes 16 patients treated for inveterate dislocation (mean 12 months; min. 2–max. 24). In 2 patients the reduction was performed (with an osteotomy of the humeral neck and with a synthesis of the fractured glenoid). Among others 14 patients there was a lesion of the humeral head that required in 7 patients a Mc Laughlin technique, a bone graft in 3 patients and a shoulder arthroplasty in 4 patients.
**Results** Patients of the group A obtained an excellent-good result in 85% of cases, with no redislocations or cephalic necrosis. In group B, we obtained a satisfactory and good result in 70% of patients. Limited ROM in abduction and external rotation is more evident in patients with total shoulder arthroplasty (in two persisted a partial posterior subluxation of the head).
**Conclusions** The traumatic posterior dislocations of the shoulder are rare, but must be diagnosed clinically for limited extrarotation and with axial X-rays and urgently reducted, or at least within the first 2 weeks to get better results. A CT scan of the humeral head is useful to evaluate the impact of the head, fracture of the glenoid or of the humeral neck and the orthopaedic can decide the most appropriate treatment. Results are influenced by the age of the patient, associated injuries, and performed rehabilitation.

### Fractures of the humeral head: our expertise in anatomical reconstruction

N. Galvano*, L. Milici, D. Martorana, M. D'Arienzo

Clinica Ortopedica, AOUP Policlinico (Palermo, IT)

**Introduction** The complex fractures of the humeral head injuries are usually difficult to be surgically treated. The goal of surgery is to restore joint function and patient autonomy in the absence of pain as quickly as possible. Is still debated, if their attitude is best in young people, rather than in the elderly ones. At the Orthopedic Clinic of the University Hospital of Palermo, in the period between 2007 and 2011, 20 cases of complex fractures of the humeral head were treated by reconstruction technique with Cage (prismatic-triangular system in titanium).

**Materials and methods** "Da Vinci System" is a titanium cage in the form of triangular-prism. Both sides are perforated to allow passage of sutures and fixation of metal, and to promote bone growth and revascularization. In the study 20 cases of fracture (fragments 3 and 4 according to the classification of Neer and Hertel) were treated with this technique. Patients were assessed by standard X-ray and CT: 12 patients had displaced fractures in 3 fragments, 6 patients had displaced fractures in four fragments, and one case had fracture-dislocation.

**Results** There were no technical or clinical complications during surgery. Patients were reassessed according to the Neer scale, taking into account above all the pain and function (100 points) at a distance of 1–3–6–12 months. The results were good (score from 90 to 100). At clinical control patients had a complete functional recovery of the shoulder. There were no cases of infection and necrosis of the humeral head.

**Discussion** Patients were evaluated on the basis of the stability of the reduction of the healing process and the appearance of complications. Patients were immobilized with a shoulder brace for 4 weeks. They started the rehabilitation of the elbow and wrist in the immediate postoperative. Any percutaneous Kirschner wire was removed after 4–6 weeks.

**Conclusions** Our results confirm that the best indication of the reconstruction technique with Cage is reserved for complex fractures of the humeral head fragments to 3–4. The reconstruction technique with Cage is a reliable procedure, leads to high patient satisfaction and a good recovery of movement, and may represent a viable alternative to both currently available techniques of osteosynthesis and the use of prostheses.

### Minimal invasive osteosynthesis for proximal humerus fractures with polarus humeral nail

M.A. Liberati, E. Crainz Fossati*, F. Di Bisceglie, M. Savoini, C. Pagliantini, D. Tigani

(Siena, IT)

**Introduction** Fractures of the proximal humerus account for 4–7% of all fractures and are relatively common especially in the elderly population in relation to increasing age and osteoporosis. The ideal treatment remains controversial topic and there is a wide variety of techniques available, particularly in patients over sixty and older, whereas 85% of the fractures, even relatively displaced, heal conservatively with satisfactory functional results.

**Materials and methods** We retrospectively reviewed 32 patients with displaced fracture of the proximal humerus (AO type A1-3, B1-3) treated with the Polarus humeral nail (Acumed), mean age 76 years (range 41–90 years), mf ratio 1:4, mean follow-up 14 months (range, 6–24) and the results were evaluated using the Constant score.

**Results** All fractures were consolidated clinically and radiographically at a mean time of 8.3 weeks (range 6–13); 88.1% of patients achieved an excellent/optimal result. Complications were: pullout proximal screws in 3 cases (4 screws), pullout distal screw in 1 case, subacromial impingement in one case. The average surgical time was 18 min (range 11–45 min).

**Discussion** The treatment of proximal humerus fractures can be very complex especially in patients over sixty, with rotator cuff tears and increased anesthetic risk. The ORIF treatment is indicated in young patients with high functional demands, but often in the elderly is hardly feasible given that we obtain good results even conservatively, albeit at the cost of a significant decrease in residual ROM.

**Conclusions** The nail Polarus proved to be an excellent option for the treatment of these fractures, allowing stable and excellent summary of results such as excellent restoration of ROM, minimally invasive, possibility of early mobilization, reduced surgical time and anesthesia, particularly in elderly patients for which remains a valid alternative to conservative treatment given the limited impact and the low rate of surgical complications.

### *C11—SHOULDER AND ELBOW 3*

### Distal biceps brachii ruptures: isokinetic comparison between reinsertion with anchors and screws

G. Castellani*[1], E. Cesari[2], D. Morici[1], A. Longhi[1], L. Sabbatini[3], M Ricci[3], A. Gigante[4]

[1]Divisione Ortopedia-Traumatologia, Ospedali Riuniti di Ancona (Ancona, IT);
[2]Istituto Humanitas (Milan, IT);
[3]Medicina Riabilitativa, Ospedali Riuniti Ancona (Ancona, IT);
[4]Clinica Ortopedica, Università di Ancona (Ancona, IT)

**Introduction** Distal biceps tendon ruptures are rare (3–4% *vs.* 96% of long head biceps ruptures). The dominant arm is mainly involved, especially in hard worker men between 30 and 60 years. The diagnosis is frequently clinical but MRI and ultrasound scan may be helpful. The reinsertion in the anatomical footprint is the gold standard in acute lesions, whilst in chronic lesions the tenodesis to brachialis muscle can be performed. When conservative treatment is administered, a loss of strength of about 40% in flexion and 30% in supination can be observed (Morrison 2002; Catoney 1995).

**Materials and methods** 35 patients (2 had bilateral ruptutre) out of 59 patients, treated from 1993 to 2010 in our Institution, were recruited for this study with a follow-up length ranging from 1 to 17 years. Three patients had conservative treatment, 20 had reinsertion in anatomical footprint using screws and 12 using suture anchors. The Henry modified approach was performed with the following steps: identification of the distal tendon stump; clamping the recurrent branch of radial artery; dissecting between brachio-radialis and pronator muscles; reinsertion of the tendon on the radial tuberosity maintaining the forearm in supination. All the patient were evaluated by X-rays and isokinetics.

**Results** There were no intraoperative complications. A neuropathy of the sensitive branch of radial nerve was observed in 1 case and in 2 cases pain in the anterior part of the elbow was reported. There were no reruptures, infections, scar problems and proximal radio-ulnar sinostosis. Etherotopic calcifications were observed in 1 patient. All the patients but one (wrestling) returned to work and sports at their preoperative levels. The 3 patients treated conservatively had strength reduction both in flexion and in supination.

**Discussion** Reinsertion in the anatomic footprint is the gold standard even if operative techniques may differ. Ware had excellent results using a single anterior incision and suture anchors (Ware 92). John et al. treated 60 patients using suture anchors obtaining excellent results in 46 and good in 7 patients at a follow-up of 38.1 months. Boyd and Anderson, proposed a combined technique with a double approach to avoid neuro-vascular risks. However, etherotopic calcifications and radio-ulnar sinostosis were observed.

**Conclusions** We believe that an anterior approach to the elbow (Henry modified) using anatomical reinsertion with suture anchors is to date the best technique to minimize neuro-vascular complications, etherotopic calcifications and radio-ulnar sinostosis.

### Glenohumeral acute instability with anterior/inferior glenoid fracture: open surgical stabilization and coracoid trasposition

M. Trono*, M. Gigli, G. Lucidi

Ospedale Infermi Rimini (Rimini, IT)

**Introduction** Anterior/inferior glenoid fracture is rare, and generally associated with acute anterior glenohumeral instability. There are several treatment options, depending on the size of the fragment. In literature, however, there is no treatment agreement. In this Abstract we present our experience with open surgical solution (screw fixation and Latarjet/Bristol coracoid transposition).

**Materials and methods** We present a series of 8 patients with anterior glenohumeral dislocation and anterior/inferior glenoid fracture, treated from 2007 to 2010 with an open surgical stabilization. In 1 case there was an associated fracture of the greater humeral tuberosity. In one case there was no shoulder dislocation but Neer 3 fracture of the proximal humerus: during surgical operation, however, an important acute anterior instability was detected. In all cases the glenoid fragment was of considerable size, extending to about 25% of the articular surface, with acute instability; thus we choose an open surgical stabilization with screws, and in 6 cases we performed an additional Latarjet/Bristol coracoid trasposition, to give better stability to the synthesis.

**Results** In all cases we obtained a good joint stability, with no recurrence of dislocation. In one case we observed partial humeral head necrosis, currently asymptomatic.

**Discussion** In all other cases not included in the study, the fragment was too small, and generated "by tearing" (bony Bankart), and nonoperative treatment was chosen. In all cases of the study a serious acute anterior instability was present, or evidence of recurrence within a few days, even inside the Deasult bandage. Therefore, the open surgical synthesis was necessary to ensure joint stability. In our point of view, the arthroscopic solution was not possible due to size and displacement of the fragments. The results present good and satisfactory functional recovery appropriate to the age of the patients. In cases of anterior dislocation with bony Bankart of medium size, treated nonoperativly, the patient should be monitored with weekly X-rays because of recurrent dislocations within inside Desault bandage, that would evolve in inveterate injury.

**Conclusions** Anterior/inferior glenoid fracture in anterior glenohumeral dislocation is a complex lesion that can cause acute instability and inveterate dislocations of the shoulder, thus it should be identified and treated appropriately. Open surgical stabilization with screws (with the additional transposition of the coracoid) can give good results.

### Humeral fractures osteosynthesis in osteoporotic patient: TGF versus K wires fixation

M. Ceretti*, S. Pappalardo, M. Fanelli

Policlinico Umberto I (Rome, IT)

**Introduction** Proximal humerus fractures are 5% of the total. The surgical treatment is indicated in 15% of cases. Osteoporosis is the first risk factor together high and low energy trauma.

**Materials and methods** The study was performed on 31 patients: 77% women, 23% men with a mean age of 77 years and a follow-up of 10 months. First diagnosis was Neer II for 6 pts, Neer III for 13 pts, Neer IV for 12 pts. Patients were also evaluated by Hertel classification to define the epifisis necrosis risk.

**Results** Free wires synthesis can reduce anatomically the fragments thanks to introduction of an indefinite amount of wires which are intra-operating bent. The free wires are also able to guarantee an early mobilization because they are introduced by retrograde way without capsular and rotator cuff damage. However if the wires are locked with clamps is possible reduce the migration risk. Obtained results demonstrate that TGF synthesis give better functional results with less risk in Neer II fractures. Therefore free wires guarantee best results in Neer IV fractures. Neer III fractures can be treated with the same results with both methods.

**Discussion** Free wires synthesis produced the right fragment re-alignment and stabilization. They help an accurate reduction in multi fragmented fractures because guarantee an early complete post-op. shoulder mobilization with a greater risk of wires migration. TGF fixation, adopted in 8 patients, reduced wire migration risk with a lower post-op. ROM caused by archlet inpingment with acromion. Complications: 3 fixation system migrations (3 K wires), 2 secondary fracture breakdowns (2 K wires), 2 infections (1 K wire; 1 TGF) and a humeral head necrosis (1 K wire).

**Conclusions** TGF reduces the free wires complications with the limit of fragments non-reduction in Neer III or more fractures. Free retrograde wires reduction guarantees an early and a large mobilization. TGF guarantees elevation and abduction post-op. movements larger than retrograde free wires. TGF has the vantage of reducing migration risk thanks to locked wires with mini invasive system. TGF offers better functional results with lower risk in Neer II fractures. Free wires synthesis guarantee better results in Neer IV fractures. Neer III fractures can be treated with both techniques with the same results.

### Shoulder replacement in the treatment of pluri-fragmentary and displaced fractures of the proximal humerus: our experience

L. Nalbone*, R. Adelfio, M. Butera, M. D'Arienzo

Clinica Ortopedica e Traumatologica, Policlinico Universitario "Paolo Giaccone" (Palermo, IT)

**Introduction** We carried out a retrospective study on the indications and the results of shoulder replacement in fractures of the proximal humerus. With reference to most classifications, shoulder replacement is the chosen treatment in complex and so-called non-reconstructable fractures of the proximal humerus.

**Materials and methods** Our study is based on 32 patients with pluri-fragmentary, displaced fractures of the proximal humerus operated with prostheses from 2004 to 2009. The age of the patients ranged from 42 to 79 years old (average 60.19). 25 patients were operated immediately (in 8 of these, the prostheses had been inplanted after an attempt at reconstruction and synthesis) and 7 for inveterate fractures.

**Results** The prostheses implanted were 28 Tournier Aequalis and 4 Tournier inversa. The follow-up ranged between 1–5 years. The results in most controlled cases showed a sufficient range of movement. In 15 patients the average active anterior elevation was about 110° and the average abduction was about 120°.

**Discussion** Shoulder replacement for acute or chronic fragmentary fractures of the proximal humerus is a procedure whose results depend on the anatomical reconstruction of the length of the humerus, on the repositioning of the tuberosities and on the appropriate orientation of the prosthesis head in relation to the glenoid. The success depends on both the surgical tecnique and an adeguate rehabilitation programme.

**Conclusions** Even if our cases are not numerous, we can conclude that shoulder replacement in cases of pluri-fragmentary, displaced fractures gives satisfactory results even if the functionality is reduced.

### The treatment of supraspinatus lesion with PRP: clinical and MRI evaluation at one-year follow-up

P. Di Benedetto*, A. Beltrame, R. Gisonni, V. Cainero, A. Causero

Clinica Ortopedica, Azienda Ospedaliero-Universitaria di Udine (Udine, IT)

**Background** If correctly executed and followed by an appropriate rehabilitation the arthroscopic repair of supraspinatus tears assures outcomes. It seems that Platelet-Rich Plasma (Prp), a concentrate of plasma rich of platelets, enhances tissue healing process thanks to the release of multiple grow tissue factors. In the last years it has been widely used in orthopaedic surgery. However, there is no certainty about advantages using this technique in rotator cuff repair. The aim of this study is the evaluation of 1-year follow-up results of arthroscopy supraspinatus repair with PrP application.

**Methods** We performed arthroscopic repair of small and medium-sized supraspinatus tendon tears in 40 patients, who were divided in 2 randomised groups. 20 patients underwent surgical repair with the application, between tendon and bone surface, of the “Cascade” membrane, obtained by high speed centrifugation of patients’ peripheral blood. A standard rehabilitation protocol for rotator cuff tears was performed. The clinical follow-up was 1-, 3-, 6-month post-surgery and MRI 1 year after surgical repair has been evaluated.

**Conclusions** Clinical improvement was achieved; MRI study provided tendon healing and fatty degeneration and muscle atrophy delayed.

### Locked posterior shoulder dislocations and fracture-dislocations: treatment options

E. Siuni*, M. Planta, F. Orgiano, I. Pusceddu

Servizio Chirurgia della Spalla SCOMR, Ospedale Marino (Cagliari, IT)

**Introduction** Posterior dislocations of the shoulder account for 2–5% of all glenohumeral dislocations. Locked posterior dislocation of the shoulder is a rare clinical entity and it is the result of missed posterior dislocation or fracture-dislocation in polytrauma patients, in young or elderly patients after epileptic seizures or high-energy trauma. The time elapsed between trauma and diagnosis and the size of the humeral head impression fracture were accepted as a useful guide for treatment of locked posterior shoulder dislocations.

**Materials and methods** We retrospectively reviewed 10 patients (4 female and 6 male; range, 23–65 years) with locked posterior shoulder dislocations or fracture-dislocations surgically treated from 2005 to 2010. In 5 cases the duration of dislocation ranged from 20 days to 1 month and the humeral head defect was about 20%. In these patients the treatment was closed reduction under general anesthesia and postoperative immobilization of the shoulder in external rotation for 4 weeks. One patient with fracture-dislocation was treated with open reduction of the dislocation, repositioning of the humeral head and interfragmentary osteosynthesis with the plate and screws. In 2 patients there was a reverse Hill-Sachs lesion between 20 and 40% and the surgical reduction was followed by a transposition of the lesser tuberosity according to the Neer modification of the Mc Laughlin procedure. Shoulder prostheses were implanted in 2 patients.

**Results** No intra-operative or post-operative complications occurred in this group of patients and stability was restored and maintained in each patient. Patients treated by closed reduction under general anesthesia reported a full recovery to their pre-traumatic physical activity level. The Constant Score was 85 points in the patient treated with the plate and screws. The 2 patients treated with Neer’s technique showed a limitation of internal rotation. In the patients treated with shoulder prosthesis the Constant scores were 68 and 56. In this last patient the score was influenced by the secondary tear of the rotator cuff.

**Discussion** The posterior dislocation of the shoulder is a rare injury that is often missed on initial observation. The locked posterior dislocation’s management depends on size of the humeral and/or glenoid defect, the duration of the dislocation and the age and activity of the patient.

**Conclusions** An early diagnosis, obtaining a good surgical treatment and functional outcomes is very important.

### Osteosynthesis of fractures of the proximal humerus plate with angular stability and mini-invasive technique: a review of our series

L. Trentani*, A. Mazzucco, S. Rossi, M.A. Gadaleta, M. Merlo

Ospedale di Circolo (Busto Arsizio, IT)

**Introduction** Although the proximal humeral fractures are common injuries, there is still a unique treatment strategy for unstable and displaced fractures (two to four-part fractures). Surgical therapy must ensure anatomic reduction and stable fixation of the fracture with minimal soft tissue and vascularization damage intraoperative in order to avoid the high risk of avascular necrosis. Procedures: indirect reduction and percutaneous fixation and intramedullary nails, in contrast with more modern techniques of fixation with fixed-angle plates, placed through minimally invasive access. To date there is an open debate on the risks and benefits of these methods.

**Materials and methods** Since 2009 we have introduced in our surgical practice, the fixed-angle plate NCB (Zimmer) for osteosynthesis in proximal humeral fractures. In 2010, the same completely replaced the use of intramedullary nails for above fractures. In this period we treated 20 patients with fractures in two, three and four fragments. All patients wore a brace for the first 15 days after surgery and have performed an outpatient rehabilitation program early. The results were evaluated with the VAS, the SST and the Constant Test at 3, 6, 12 months.

**Results** The results at 3 months were satisfactory-good in 80% of cases. However, there was a progressive improvement of the same after three and six months, with the progression of functional rehabilitation, which for some patients was delayed. We had no cases of injury circumflex nerve, but in 3 cases the patients had a stupor, whit regression at 3 and 6 months.

**Discussion** The approach to patients with fractures of the proximal humerus at present exceed the outdated concept of percutaneous treatment, which exposes the patient to long periods of postoperative immobilization, or with intramedullary nails, which especially in middle-aged patient will damage to the rotator cuff often intact. The introduction of new procedures of osteosynthesis, which can be used with little damage to the soft tissues, can handle a wide age range and an equally broad spectrum of lesions, without increasing the risks inherent in procedure.

**Conclusions** The treatment of displaced and several fragments fractures of the proximal humerus with NCB-PH plates provides superior interfragmentary stability and good restoration of function, also in elderly patients with good functional requirements and good compliance to the rehabilitation treatment. Nevertheless the use of plates angular stability require a surgical procedure that respects the anatomy and allow a rapid mobilization.

### *C12—SHOULDER AND ELBOW 4*

### Pathoanatomy of soft tissue constraints in complex elbow instability and reconstruction results with suture anchors

G. Giannicola*, D. Polimanti, F.M. Sacchetti, E. Manauzzi, G. Bullitta

Università di Roma “Sapienza” (Rome, IT)

**Introduction** The aim of our study was to analyze prospectively in a large group of patients with complex elbow instability (CEI): (1) the frequency and pathoanatomy of soft tissue constraints, and (2) clinical results of ligament repairs with suture anchors.

**Materials and methods** From 2005 to 2009 the same surgeon operated 47 patients with CEI. Lateral compartment injuries were identified by direct visualisation in all cases. MCL tear was inferred through intraoperative valgo-pronation stress testing/fluoroscopy when not addressed surgically (41 pts). The ligament injuries were divided into two categories: simple and complex lesions. *Simple lesions*: (1) isolated proximal avulsion; (2) isolated distal avulsion, associated or not with a bone fragment; (3) isolated mid-substance lesions. *Complex lesions* consisted of two or more simple types of lesion. Postero-lateral joint capsule (PLC) injuries were defined as Minor PLC Lesions if there was a small detachment (<1 cm) by the postero-lateral aspect of distal humerus adjacent to LCL origin, and Major PLC Lesions when the detachement was greater than 1 cm. The common extensor origin (CEO) and flexor-pronator origin (FPO) injury was defined as present if a lack of continuity of more than half of the muscle tendon complex was observed. In 31 out of 47 patients, the repair of capsule-ligamentous structures was carried out using double-wire suture anchors. The mean follow-up was 25 months. MEPS, ASES and DASH scores were used for outcome evaluation.

**Results** Proximal LCL avulsion was the most common pattern of injury (31 cases). In these cases, it was always associated with a PLC lesion classified as major in 26 % of cases. Proximal LCL avulsion was associated with mid-substance tear in 71% of cases. Distal and mid-substance ruptures of LCL were observed in 8 and 13% of 47 patients, respectively. A rupture of CEO was present in 21% of patients, while MCL was torn in 45%. Elbow stability was obtained in 97% of patients after surgery. A residual mild instability was observed in four cases and did not affect the clinical results. The average MEPS score was 93; the average DASH 7.6; the average ASES 88.

**Discussion** The accurate identification of the presence and pathoanatomy of soft tissue injuries is essential to the planning of correct surgical treatment to obtain recovery of joint stability. After ORIF, ligament reconstruction with suture anchors leads to satisfactory functional results in most patients with CEI.

### Anatomical variations of the proximal radius: implications in osteosynthesis with plate

G. Giannicola*, E. Manauzzi, A. Greco, F.M. Sacchetti, G. Bullitta

Sapienza Università di Roma (Rome, IT)

**Introduction** Some Mason Type II and all Mason III fractures are known to be complex injuries of the radial head (RH) in which results of treatment are often unpredictable, especially in fractures with neck comminution. This is related to the difficulties in achieving a stable fixation and in restoring the cervico-cephalic angles and the medial offset of the RH. Recently osteosynthesis with precountered plate has been proposed as a way to improve results. Several studies have analyzed the anatomical landmarks of the safe zone for correct placement of osteosynthesis devices in radial head and neck fractures. However, no study has analyzed whether currently used plates adequately match the profile of the proximal radius in the “safe zone”. In the present investigation, a morphometric study of the proximal radius was conducted to evaluate the morphologic aspects of the safe-zone and the congruence between the proximal radius and a currently used plate.

**Materials and methods** Forty-four dried radial cadaveric bones were analyzed. The entire length of the radius, height of neck and head, minimum and maximum diameter of radial head were measured. The morphologic aspect of the cervico-cephalic curvature of the safe-zone and the plate-congruence were evaluated qualitatively and quantitatively.

**Results** Three different morphologic variations of the safe zone with significant differences in the bending radius were found: Types A, B and C showing a flat (25%), a low concave (64%) and a marked concave (11%) curvature, respectively. Morphologic Types A and C exhibited poor and moderate congruence, respectively. Morphologic type B, which better matched the profile of the plate, showed a good, moderate or poor congruence in 42.9, 50 and 7.1% of cases, respectively. The bending radius was found to be independent of radial dimensions.

**Discussion** We identified three morphologic Types of the safe zone with different concavities; such a difference should be taken into account in order to achieve an osteosynthesis of the radial head which properly reconstitutes the anatomy of the proximal radius. As the profile of the proximal radius in the safe zone was found to show substantial morphologic variations, a single plate design is not sufficient to perform proper ORIF in all these complex fractures. We suggest that a pre-operative radiograph of the contra-lateral uninjured radius should be taken in comminuted radial head and neck fractures, in order to select the most appropriate plate to restore the original morphology of the proximal radius.

### The use of absorbable pins (of poly-p-dioxanone) in displaced articular radial head fractures of 53 patients

C. Rovesta*, G. Gazzotti, G. Leo, S. Colopi, M. Francia, G. De Luise

(Modena, IT)

**Introduction** In displaced articular radial head fractures (Mason 2–3), osteosynthesis can be achieved with plates or screws in the “safe zone”. Since 2006 we have used absorbable poly-p-dioxanone pins that can be inserted over the whole surface of the radial head.

**Materials and methods** From July 2006 to September 2010 at the Orthopedic Clinic of Modena, we used absorbable pins (2 pins in 22

cases, 3 in 18 cases, 4 in 9 cases, 5 in 2 cases, 6 in 2 cases) in 53 patients (28 male, 25 female; mean age, 45 years; range, 20–72 years) with displaced articular radial head fractures (classified as Mason 2–3). Functional outcomes were evaluated with Mayo score, DASH questionnaire and X-rays. In cases of particular pain or not clear functional limitation, we performed MRI to confirm the fracture consolidation, articular surfaces regularity, bone reaction and pins resorption.

**Results** All fractures achieved a consolidation, and most of patients presented good clinical and functional results (follow up: min-max 4–50 months). In elderly patients a minimal pain and deficit of ROM in the last degrees of pronation and supination was highlighted. We did not identify cases of intolerance of pins and it was never necessary to remove them. Magnetic resonance imaging confirmed adequate strength during critical early stages on healing and the reabsorption of the pins in an averaged time of 24 months.

**Discussion** Our clinical and radiographic results are comparable with those obtained in the literature using normal or headless compression screws, but with possibility to be used also in no safe zone. Pins can also been introduced at different levels with better grip of the fragments increasing stability of the fracture and allowing an early mobilization.

**Conclusions** The use of absorbable poly-p-dioxanone pins allows the treatment of displaced articular radial head fractures Mason 2–3. The advantages of this synthesis are the ease of application, adequate strength and good tolerability.

### Displaced mid-shaft clavicular fractures: surgical treatment with a pre-contoured angular stability plate

G. Campochiaro*, C. Tsatsis, G. Gazzotti, M. Rebuzzi, F. Catani

(Modena, IT)

**Introduction** The treatment of displaced mid-shaft clavicle fractures is highly controversial. In the last years, several biomechanical studies showed better functional results after surgical treatment. Purpose of this study is to evaluate the use of pre-contoured angular stability plate in this type of fracture.

**Materials and methods** From June 2005 to July 2009 we surgically treated 89 patients with displaced clavicle fracture. We reviewed 68 patients for a total of 70 clavicles. Functional outcomes were assessed with Constant score, Dash questionnaire; X-ray. The Mean follow-up was 2 years.

**Results** Excellent and good results were achieved in all the patients reviewed. The mean Costant score was 94.1 pt and DASH questionnaire was 4.1. We had two cases of nonunion, no case of infection and vascular or nervous lesions.

**Discussion** A review of the international literature indicates that there is not a widely accepted gold standard for treatment of displaced mid-shaft clavicular fractures. In the last 10 years biomechanical and clinical studies have shown that nonoperative treatment for severe comminution and marked shortening of the clavicle longer than 2 cm, may result in lower functional outcomes or higher percentage of nonunion. Evaluating advantages and disadvantages, our clinical experience suggests the use of pre-contoured angular stability plate in the surgical treatment of these fractures.

**Conclusions** Nowadays numerous surgical options are available for treatment of displaced mid-shaft clavicular fractures. Our experience with pre-contoured angular stability plates has shown excellent clinical results. On the basis of our study we support the use of pre-contoured angular stability plate.
